# The role of *Sp140* revealed in IgE and mast cell responses in Collaborative Cross mice

**DOI:** 10.1172/jci.insight.146572

**Published:** 2021-06-22

**Authors:** Kazufumi Matsushita, Xin Li, Yuki Nakamura, Danyue Dong, Kaori Mukai, Mindy Tsai, Stephen B. Montgomery, Stephen J. Galli

**Affiliations:** 1 Department of Pathology, Stanford University School of Medicine, Stanford, California, USA.; 2 Department of Immunology, Hyogo College of Medicine, Nishinomiya, Japan.; 3 Department of Genetics, Stanford University School of Medicine, Stanford, California, USA.; 4 CAS Key Laboratory of Computational Biology, CAS-MPG Partner Institute for Computational Biology, Shanghai Institute of Nutrition and Health, University of Chinese Academy of Sciences, Chinese Academy of Sciences, Shanghai, China.; 5 Sean N. Parker Center for Allergy and Asthma Research, Stanford University School of Medicine, Stanford, California, USA.; 6 Department of Microbiology and Immunology, Stanford University School of Medicine, Stanford, California, USA.

**Keywords:** Immunology, Inflammation, Allergy, Genetic variation, Mast cells

## Abstract

Mouse IgE and mast cell (MC) functions have been studied primarily using inbred strains. Here, we (a) identified effects of genetic background on mouse IgE and MC phenotypes, (b) defined the suitability of various strains for studying IgE and MC functions, and (c) began to study potentially novel genes involved in such functions. We screened 47 Collaborative Cross (CC) strains, as well as C57BL/6J and BALB/cJ mice, for strength of passive cutaneous anaphylaxis (PCA) and responses to the intestinal parasite *Strongyloides venezuelensis* (*S*.*v.*). CC mice exhibited a diversity in PCA strength and *S*.*v.* responses. Among strains tested, C57BL/6J and CC027 mice showed, respectively, moderate and uniquely potent MC activity. Quantitative trait locus analysis and RNA sequencing of BM-derived cultured MCs (BMCMCs) from CC027 mice suggested *Sp140* as a candidate gene for MC activation. siRNA-mediated knock-down of *Sp140* in BMCMCs decreased IgE-dependent histamine release and cytokine production. Our results demonstrated marked variations in IgE and MC activity in vivo, and in responses to *S*.*v.*, across CC strains. C57BL/6J and CC027 represent useful models for studying MC functions. Additionally, we identified *Sp140* as a gene that contributes to IgE-dependent MC activation.

## Graphical abstract

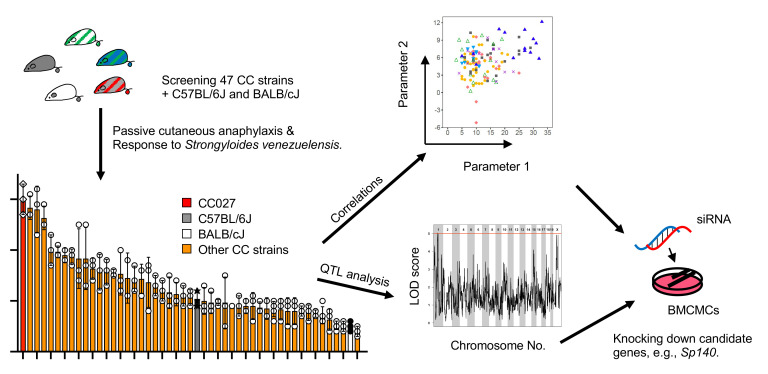

## Introduction

Mast cells (MCs) are of hematopoietic origin but reside in almost all vascularized tissues (1, 2). MCs play a pivotal role in type-2 inflammation (1, 2) and, in concert with IgE, help to protect mice from animal venoms (3), infections with *Staphylococcus aureus* (4), and infections with certain parasites (5). On the other hand, the IgE-MC pathway is well recognized as a cause of pathology in the context of allergic disorders (1, 2, 6). MCs have also been implicated in diseases not typically associated with type-2 immunity, including autoimmunity (7, 8) and cancer (9, 10). Importantly, the MCs’ role in such settings often depends on the disease model and the host’s genetic background (8–17). Therefore, elucidating how MC phenotypes and functions can vary in different genetic backgrounds is critical in efforts to understand the potential roles of MCs in health and disease.

Inbred laboratory mouse strains, such as C57BL/6J and BALB/cJ, have been widely used to uncover MC functions in vivo (18). Indeed, much of our knowledge regarding the mammalian immune system and related pathology is based on the results obtained from mouse studies, particularly those involving mutations on the C57BL/6J background (19, 20). However, because humans are genetically diverse, using limited mouse strains may not fully capture the diversity of human MC phenotypes and MC-related diseases. In addition, we don’t know to what extent the traditionally used mouse strains represent the MC phenotypes to be found among the entire *Mus musculus* species (18). It is even possible that some genes importantly involved in MC development/function could have been overlooked by studies conducted using classical laboratory strains.

Collaborative Cross (CC) mice are a reproducible yet genetically diverse genetic reference panel derived from 8 founder strains representing major *Mus musculus* subspecies (*M*.*m*. *musculus*, *M*.*m*. *domesticus*, and *M*.*m*. *castaneou*): 5 classical inbred strains (A/J, C57BL/6J, 129S1/SvImJ, NOD/LtJ, and NZO/H1LtJ), and 3 wild-derived strains (CAST/EiJ, PWK/PhJ, and WSB/EiJ) (21, 22). These strains are thought to capture about 90% of the genetic variation among the commonly used strains of laboratory mice, and the variation randomly distributes across the genome. CC mice, therefore, can be used to better capture the breadth of physiological or pathological phenotypes caused by genetic variation, to identify the influence of genetic polymorphisms (23–25), and to establish better mouse models to reflect human disease pathology (25–27).

In this study, we screened 47 CC strains, in addition to classical C57BL/6J and BALB/cJ mice, for the strength of passive cutaneous anaphylaxis (PCA). PCA is an experimental anaphylactic reaction that is dependent on IgE and MC and, therefore, reflects — at least in part — the strength of MC degranulation in vivo (28). We also evaluated infections with the intestinal parasite *Strongyloides venezuelensis* (*S*.*v.*), because IgE and MCs partially contribute to the primary immune response to this nematode (5). The aims of our study were (a) to understand the phenotypic range of PCA reactions in mice (and how “representative” PCA reactions are in C57BL/6J and BALB/cJ strains); (b) to discover strains with phenotypes useful for establishing new models for analyzing IgE and/or MC functions; and (c) to identify novel genes or genetic loci affecting MC functions.

## Results

### All immunological parameters examined are widely diverse across CC strains.

**Figure 1:**
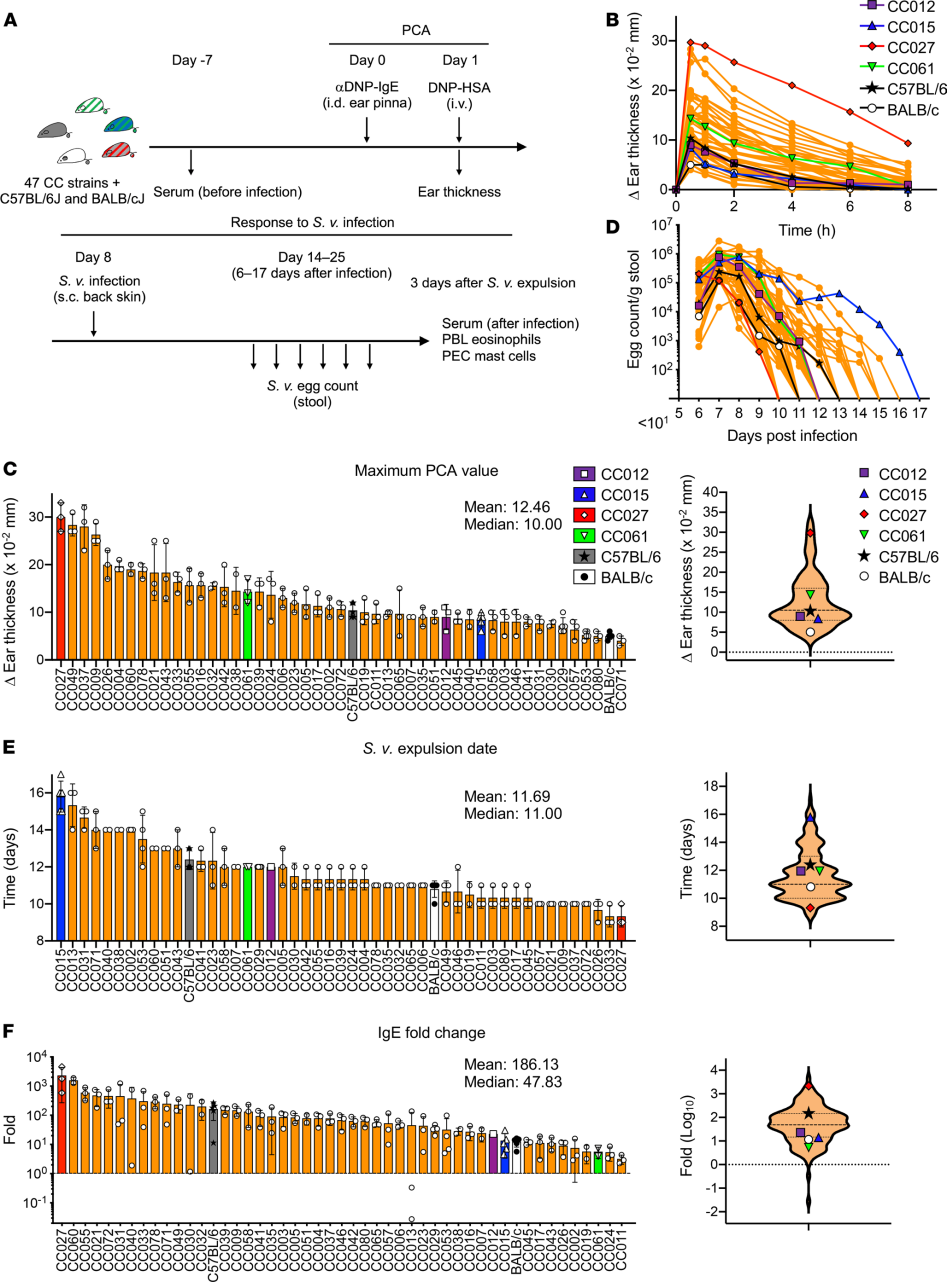
PCA intensity, duration of *S.v.* infection, and IgE fold change were assessed in CC, C57BL/6J, and BALB/cJ mice. (**A**) Schematic representation of screening using CC mice. (**B**) PCA reactions induced by injections of anti–DNP IgE and DNP-HSA. Mean changes (Δ) in ear thickness over time after i.v. injection of DNP-HSA in each genotype of mice. (**C**) Summary graphs (left) and violin plots (right) of the maximum values of PCA. (**D**) *S.v.* infections. Mean *S.v.* egg numbers excreted after worm inoculation in each mouse genotype. (**E** and **F**) Summary graphs (left) and violin plots (right) of *S.v.* expulsion date (**E**) and IgE fold change (**F**) of each mouse genotype. *n =* 1–5 mice per genotype (Supplemental Table 1) from 8 independent experiments. Data are shown as mean ± SD, and each dot indicates 1 mouse. Dashed lines in violin plots indicate 25th and 75th quartiles and median.

We screened 47 CC strains, and conventional C57BL/6J and BALB/cJ mice, for the strength of PCA reactions and the responses to a primary infection with *S*.*v.* (Figure 1A). In murine rodents, PCA reactions are relatively weak in young animals, in part because of limited MC maturation (29, 30). Therefore, all of the 153 mice used were 22–35 weeks old before starting the experiments.

We bled mice 1 week before the beginning of the PCA (day –7) to evaluate baseline levels of serum IgE and IgG1 (Supplemental Figure 1; supplemental material available online with this article; https://doi.org/10.1172/jci.insight.146572DS1). CC mice exhibited marked variation in their baseline levels of IgE and IgG1, and we found significant quantitative trait loci (QTLs) for baseline IgE and IgG1 on chromosome 10 and 12, respectively (Supplemental Figure 2).

Mice then were passively sensitized with IgE by intradermal injection of mouse anti-dinitrophenyl (anti-DNP) IgE (day 0) into the right ear pinnae, with vehicle alone injected into the left ear pinnae. A day (i.e., 24 hours) later (day 1), mice were i.v. injected with anti-DNP–human serum albumin (HSA), and mouse ear thickness was measured.

On day 8 (i.e., 7 days after PCA), the mice were infected with *S*.*v.* by s.c. injection of 5000 L3 larvae. The number of *S*.*v.* eggs in the stools was counted every day from day 6 after infection (day 13) until the mice stopped producing eggs. Three days after the mice stopped producing *S*.*v*. eggs, mice were sacrificed. We then collected peritoneal cells and blood samples to evaluate the number of peritoneal MCs and peripheral blood eosinophils, as well as the levels of serum IgE and IgG1 (Figure 1A).

The screening of 47 CC strains, together with C57BL/6J and BALB/cJ mice, showed a wide range of intensity of PCA reactions (in response to in vivo IgE-dependent MC activation) across the mouse strains (Supplemental Table 1). Most of the mice showed the highest PCA value (PCA MAX) at 30 minutes after antigen injection, and the rest of the mice had a PCA MAX at 1 hour after the injection (Figure 1B). The PCA MAX varied from 4 × 10^–2^ to 33 × 10^–2^ mm, with an average of 12.46 × 10^–2^ mm and a median of 10.00 × 10^–2^ mm (Figure 1C).

C57BL/6J mice exhibited an average PCA MAX of 10.4 × 10^–2^ mm, which was close to the median value for all strains (specifically, 24th out of 49 strains). This indicates that C57BL/6J is a reasonable “average” mouse model for studying mouse IgE–dependent MC activation in vivo. By contrast, BALB/cJ mice showed an average PCA MAX of 5 × 10^–2^ mm, the second lowest among the strains, indicating that this strain had a remarkably weak PCA reaction in the naive state.

The duration of *S*.*v*. infection in the different mice varied from 9 to 17 days (Figure 1, D and E). Interestingly, CC027 mice, which showed the strongest PCA MAX, expelled *S*.*v*. in the shortest period (Figure 1E). C57BL/6J mice stopped *S*.*v*. egg production at 12.4 days after infection, 38th among the 49 strains, and BALB/cJ mice stopped egg production at 10.8 days (17th among the 49 strains), probably reflecting the well-characterized Th1 versus Th2 skewing characteristics of these 2 strains (31).

Serum IgE and IgG1 levels after *S*.*v*. infection, the fold changes of the antibody levels, the percentages of eosinophils in peripheral blood leukocytes (PBL), and peritoneal MC numbers also varied widely across the strains (Figure 1F and Supplemental Figure 3). Interestingly, CC027 mice, the strain that showed the highest PCA intensity and the earliest *S*.*v*. expulsion, also showed the highest IgE fold change (Figure 1F) and PBL eosinophil percentage (Supplemental Figure 3D). This suggests that this strain has a remarkably strong type-2 immunological response. Notably, all of the screened parameters from C57BL/6J mice, but not from BALB/cJ mice, were moderate and most of them were in the interquartile range (Figure 1, C–F, and Supplemental Figure 3); C57BL/6J’s IgE-fold change was slightly above the 25th quartile, at 164.2 versus 145 (Figure 1F).

To validate the accuracy of the screening results, we repeated the experiments using a limited number of CC strains (CC012, CC015, CC027, and CC061) together with C57BL/6J and BALB/cJ mice (Supplemental Figure 4). Although all of these mice showed PCA values that were relatively weaker than in the screening experiment, probably because they were younger (14–16 weeks old) than the mice used for the initial screening, the strains’ characteristics were consistent with those detected in the initial screening. For example, CC027 mice had the strongest PCA MAX and IgE fold change and the shortest time for *S*.*v*. expulsion, CC015 mice had the longest *S*.*v*. infection, and BALB/cJ mice had the lowest PCA value.

**Figure 2:**
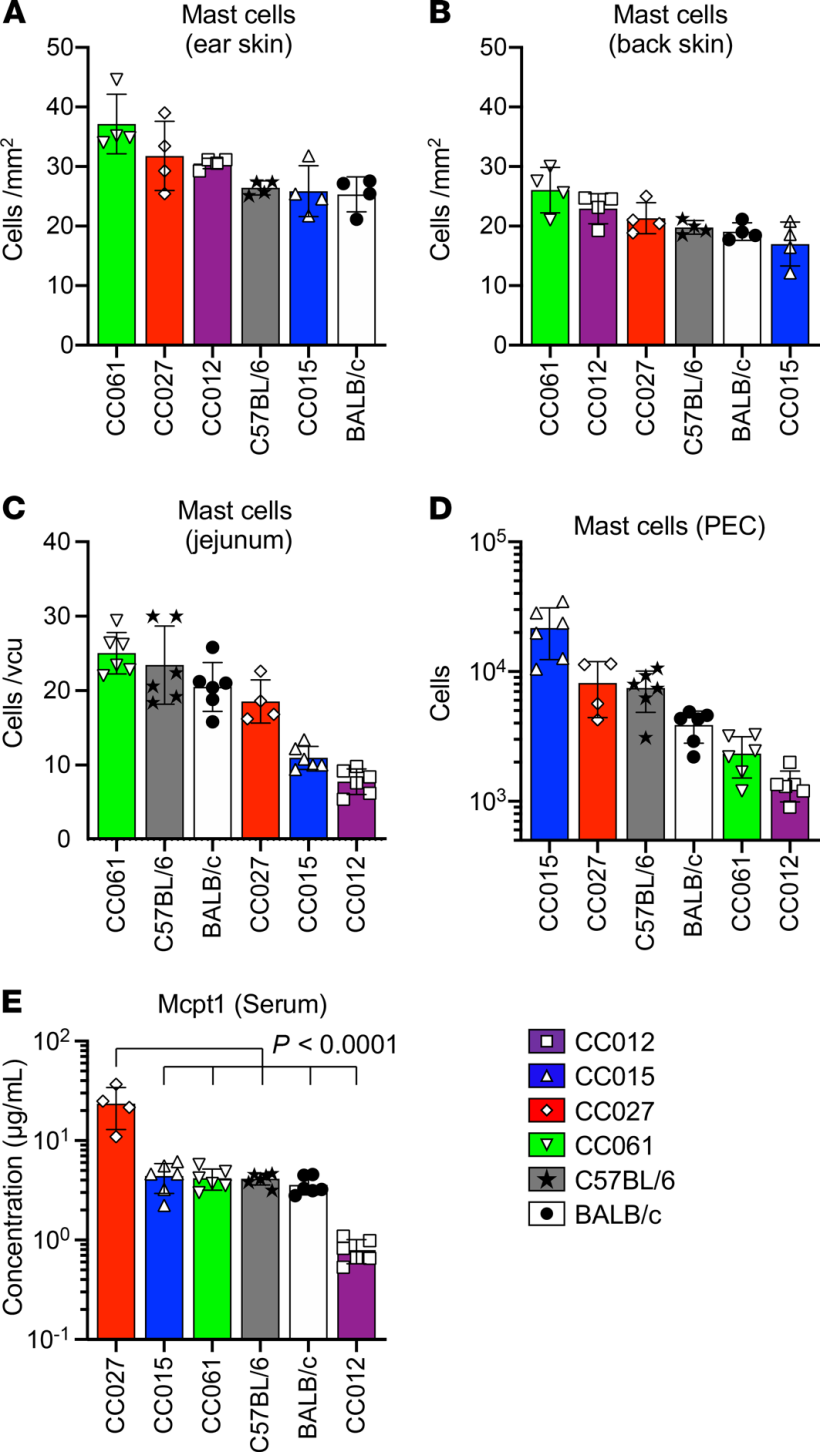
Tissue MC numbers and serum Mcpt1 levels were evaluated in CC, C57BL/6J, and BALB/cJ mice. CC012, CC015, CC027, CC061, C57BL/6J, and BALB/cJ mice were treated as in Figure 1A. (**A**–**E**) MC numbers in ear (**A**) and back skin (**B**) from naive mice, as well as MC numbers per villus crypt unit (vcu) in jejunum (**C**) and peritoneal exudate cells (PEC) (**D**), and serum Mcpt1 levels (**E**) from *S.v.*-infected mice 3 days after the mice stopped producing *S.v.* eggs. Data are shown as mean ± SD, and each dot indicates 1 mouse. *n =* 4 for **A** and **B** and CC027 in **C**–**E** and *n =* 6 for other genotypes in **C**–**E** from 2 (**A** and **B**) and 3 (**C**–**E**) independent experiments.

We also evaluated the numbers of MCs in ear and back skins (Figure 2, A and B). The MC numbers did not differ as much among the strains as did the intensities of PCA. Furthermore, MC numbers in the jejunum (Figure 2C) and peritoneal exudate cells (PEC) (Figure 2D) 3 days after *S*.*v*. expulsion did not correlate with resistance against *S*.*v*. However, the CC027 mice, which showed the highest PCA intensity and the earliest *S*.*v*. expulsion, had the highest levels of serum mouse MC protease 1 (Mcpt1; Figure 2E). Therefore, the differences observed in the strengths of the PCA reactions and responses to *S*.*v*. in the various strains did not correlate strongly with differences in MC numbers but, instead, appeared to correlate with the extent of MC activation.

### PCA intensity, duration of S.v. infections, and the IgE fold change correlate in CC mice.

**Figure 3:**
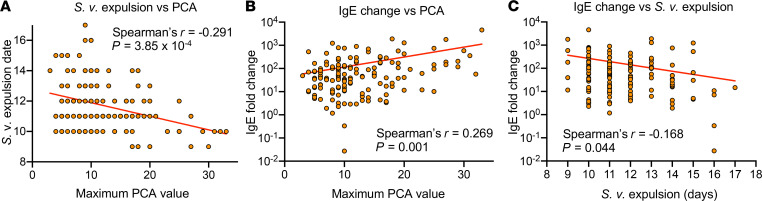
PCA intensity, duration of *S.v.* infection, and IgE fold change correlate in CC mice. (**A**–**C**) Maximum PCA value (*x* axis) relative to *S.v.* expulsion date (*y* axis) (**A**), maximum PCA value (*x* axis) relative to IgE fold change (*y* axis) (**B**), and *S.v.* expulsion date (*x* axis) relative to IgE fold change (*y* axis) (**C**). Each dot indicates a value for 1 mouse. The respective Spearman’s rank-order correlation coefficients (*r*) and *P* values for each analysis are shown.

Because the variety of values obtained by screening among the strains was much wider than that obtained within each strain, we reasoned that all of the screened parameters were likely to be heritable traits. Therefore, we next examined the correlations among the screened parameters. Spearman’s rank correlation indicated that PCA MAX weakly but significantly correlated with the brevity of *S*.*v*. infection (*P =* 3.85 × 10^–4^), the magnitude of the IgE fold change (*P =* 0.001), and the number of PBL eosinophils (*P =* 0.0318), but with lower numbers of peritoneal MCs (*P =* 2.20 × 10^–4^; Figure 3 and Supplemental Table 2). In addition to correlating significantly with the PCA MAX, the brevity of *S*.*v*. infection also correlated with the IgE fold change (*P =* 0.0438) and the postinfection IgG1 levels (*P =* 0.0311; Supplemental Table 2). Therefore, the IgE fold change was the only parameter tested that correlated significantly with both PCA MAX and the brevity of *S*.*v*. infection, and these 3 parameters were the only multiple parameters tested that were mutually correlated with one another (Figure 3).

### Genome-wide association analysis identifies regions contributing to MC functions.

Given that the PCA MAX, a shorter duration of *S*.*v*. infection, and the magnitude of the IgE fold change correlated with one another (Figure 3), we hypothesized that the 3 traits might reflect the effects of the same genetic locus (or loci). We, therefore, performed QTL analyses for each of them (Supplemental Table 3).

**Figure 4:**
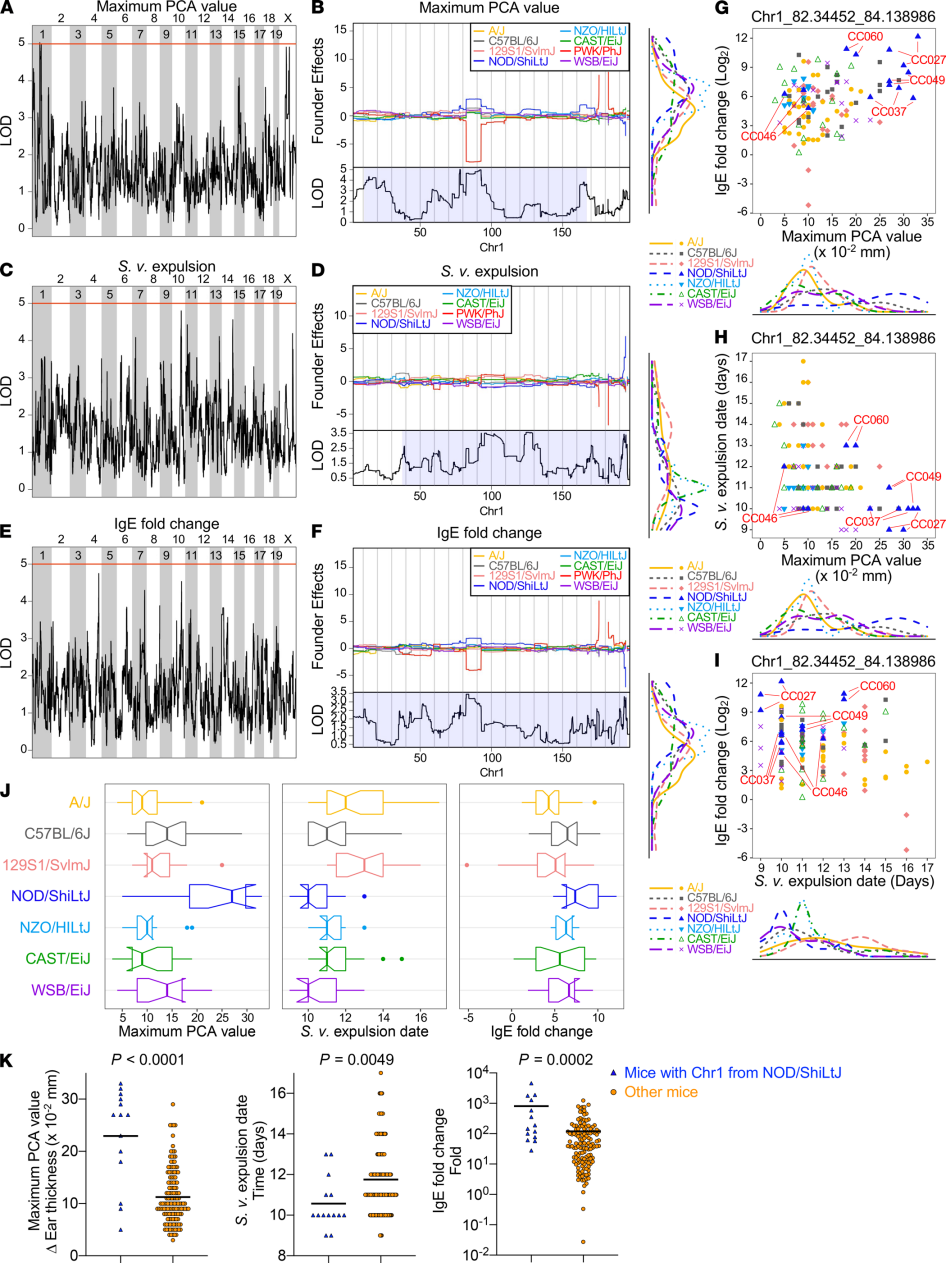
Chromosome 1, region 81.7–92.9 Mb, from NOD/ShiLtJ associates with stronger MC responses in CC mice. (**A**, **C**, and **E**) QTL analysis for chromosomal regions associated with PCA values (**A**), *S.v.* expulsion date (**C**), and IgE-fold change (**E**). The red lines indicate the LOD score threshold for *P =* 0.05. (**B**, **D**, and **F**) Analysis for founder effects associated with the PCA values (**B**), *S.v.* expulsion date (**D**), and IgE-fold change (**F**) within chromosome 1. (**G**–**I**) Intercorrelation among maximum PCA value, *S.v.* expulsion date, and IgE-fold change in CC mice as in Figure 2. The founder strain of each CC mouse’s chromosome 1, 82.3–84.1 Mbp, is indicated in 7 different colors. (**J** and **K**) CC mice were classified into 7 groups according to their founders of chromosome 1, 82.3–84.1 Mbp (**J**), or divided into 2 groups according to their chromosome 1, 82.3 to 84.1 Mbp, was from NOD/ShiLtJ or not (**K**). Notched box plots (**J**) and dot plots (**K**) of maximum PCA value, *S.v.* expulsion date, and IgE-fold change of each group are shown. Each dot indicates 1 mouse. Data are shown as mean ± SD. *P* values were determined by Student’s *t* test.

For PCA MAX, we identified a weak but significant QTL (*P =* 0.0031) on chromosome 1 in the interval of 81.7–92.9 Mb (Figure 4A), with a clear contribution of a founder haplotype in the NOD/ShiLtJ strain (Figure 4B). The duration of *S*.*v*. infection and IgE fold change did not show any significant peaks (Figure 4, C and E), but we found a weak contribution of the chromosome 1, region 81.7–92.9 Mb, to the 2 traits, and the causal founder haplotype variant for both traits was also of the NOD/ShiLtJ strain (Figure 4, D and F).

Figure 4, G–I, shows the effects of the founder strains of chromosome 1, region 81.7–92.9 Mb, in each mouse in the interactions among PCA MAX, the brevity of *S*.*v*. infection, and IgE fold change. Mice that had a NOD/ShiLtJ strain–derived chromosome 1, region 81.7–92.9 Mb (CC027, CC037, CC046, CC049, and CC060), were concentrated in the high PCA and short duration of *S*.*v*. infection, high PCA and high IgE fold change, and high IgE fold change and short duration of *S*.*v*. infection groups. Indeed, the values of PCA MAX, date of *S*.*v*. expulsion, and IgE fold change were significantly different between the mice with NOD/ShiLtJ strain–derived chromosome 1, region 81.7–92.9 Mb, and the other mice (Figure 4, J and K). These results strongly suggested that chromosome 1, region 81.7–92.9 Mb, derived from the NOD/ShiLtJ strain, positively influences both PCA results and the mouse response to this parasite infection.

Within 81.7–92.9 Mb on chromosome 1, we found 100 protein-coding genes (Supplemental Table 4). A search of the Sanger database (24, 32, 33) revealed that only 4 genes — *Col4a4*, *Col4a3*, *Sp100*, and *Sp140* — had NOD/ShiLtJ strain–specific protein–changing SNPs (missense or splice region variants). Of these, *Sp140* seemed to be the most plausible candidate, as it is reported to encode a nuclear factor that contributes to the expression of a set of inflammatory genes in LPS-stimulated macrophages (34). In addition, the SNPs on *Sp140* associate with autoimmune diseases, such as Crohn’s disease (34–36) and multiple sclerosis (37), in humans, where MCs have been proposed to potentially contribute to the pathogenesis (38–41).

We also explored potential QTLs negatively affecting MC functions. This revealed that chromosome 10 in the interval of 79.4–94.0 Mb from the NZO/HILtJ strain contributed to a lower PCA MAX, a longer duration of *S*.*v*. infection, and lower IgE fold change (Supplemental Figure 5). We found 231 protein-coding genes within 79.4–94.0 Mb on chromosome 10 (Supplemental Table 4). A search of the ENCODE database revealed that 4 genes — *Zbtb7a*, *Polr3b*, *Cfap54*, and *Usp44* — had NZO/HILtJ strain–specific protein–changing SNPs (missense variants or in-frame insertion).

### CC027 MCs show strong IgE-dependent activation in vitro.

The results discussed above indicate that CC mice with an NOD/ShiLtJ strain–derived chromosome 1 in the interval of 81.7–92.9 Mb have intense PCA activity in vivo. One possibility to explain such a phenotype is more robust IgE-dependent MC activation. To examine if this might also be observed in MCs cultured in vitro, we generated BM-derived cultured MCs (BMCMCs) from 4 CC mouse strains (CC012, CC015, CC027, and CC061) in addition to from C57BL/6J and BALB/cJ mice.

**Figure 5:**
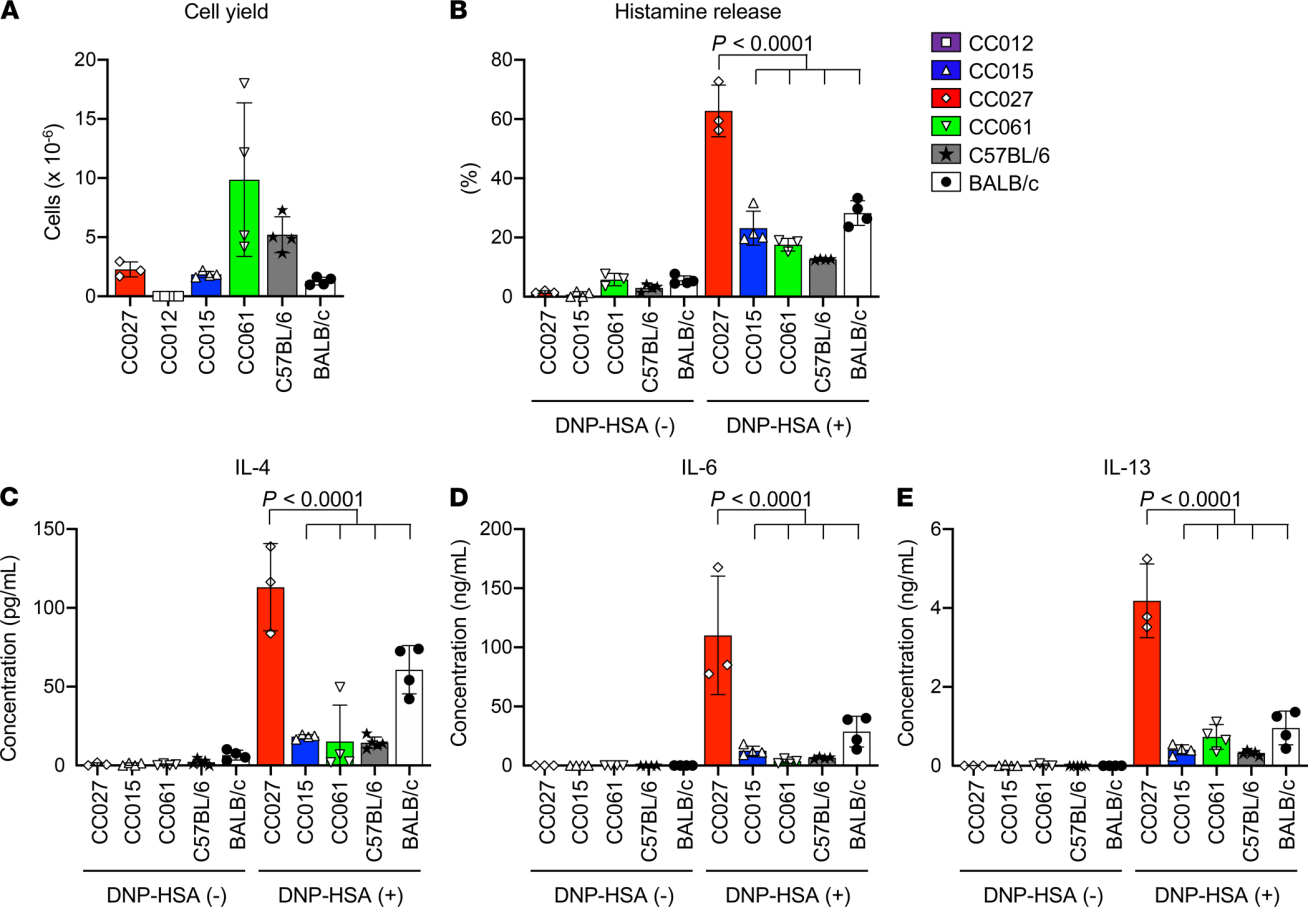
MCs from CC027 mice show strong in vitro IgE-dependent activation. BM cells from CC012, CC015, CC027, CC061, C57BL/6J, and BALB/cJ mice were cultured with IL-3 for 6–7 weeks to generate BMCMCs. (**A**) MC numbers at 6 weeks of culture were determined by FACS. (**B**) MCs were sensitized with mouse anti–DNP IgE mAb (1 μg/mL) overnight, then stimulated with DNP-HSA (20 ng/mL) for 1 hour. Histamine contents in cell lysates and the culture supernatants were determined by ELISA. Histamine release rates are indicated as percent release (supernatants) out of total histamine content of the cells (cell lysate plus supernatant). (**C**–**E**) MCs were stimulated as in **B** but for 24 hours. The levels of IL-4 (**C**), IL-6 (**D**), and IL-13 (**E**) in the culture supernatants were determined by ELISA. Data are shown as mean ± SD, and each dot indicates different biological replicates. *n =* 3–4 in each genotype pooled from 2 independent experiments. *P* values were determined by 1-way ANOVA followed by Tukey’s post hoc test.

CC027 mice exhibited the strongest PCA reaction, the highest IgE fold change, and the shortest duration of *S*.*v*. infection (Figure 1), and they have a NOD/ShiLtJ strain–derived chromosome 1 in the interval of 81.7–92.9 Mb (Figure 4). BM cells from CC027 and the 5 other strains of mice were cultured in the presence of IL-3 for 6–7 weeks. Cell yields varied among the strains. Interestingly, BM cells from CC012 mice did not produce any BMCMCs under such culture conditions (i.e., with IL-3) (Figure 5A). CC012 mice also showed the lowest jejunal and peritoneal MC numbers and serum Mcpt1 levels after *S*.*v*. infection (Figure 2, C–E, and Supplemental Figure 3E), and these results might be partially explained by the BM cells’ weak response to IL-3 (Figure 5A).

Using the BMCMCs derived from the other 5 strains, we examined their IgE-induced responses. BMCMCs from CC027 mice showed the highest percentage of histamine release among the strains (Figure 5B), consistent with CC027’s strong PCA reactions in vivo (Figure 1, B and D). CC027 strain–derived BMCMCs also produced the largest amounts of IL-4, IL-6, and IL-13 in response to IgE ligation (Figure 5, C–E). Therefore, BMCMCs derived from CC027 mice have a notably strong IgE-induced response in vitro, further suggesting that NOD/ShiLtJ strain–derived chromosome 1 in the interval of 81.7–92.9 Mb positively controls IgE-dependent MC activation.

To gain more insight into how this genetic region might control IgE-dependent MC activation, we compared the gene expression profiles of BMCMCs from CC027 and C57BL/6J mice. RNA sequencing (RNA-seq) was performed with BMCMCs from CC027 and C57BL/6J mice in the presence or absence of IgE ligation for 1 hour. The result showed 1259 upregulated and 1669 downregulated genes in the resting state, and 1784 upregulated and 2053 downregulated genes after IgE ligation, between CC027 and C57BL/6J BMCMCs (Supplemental Figure 6, A and B).

**Figure 6:**
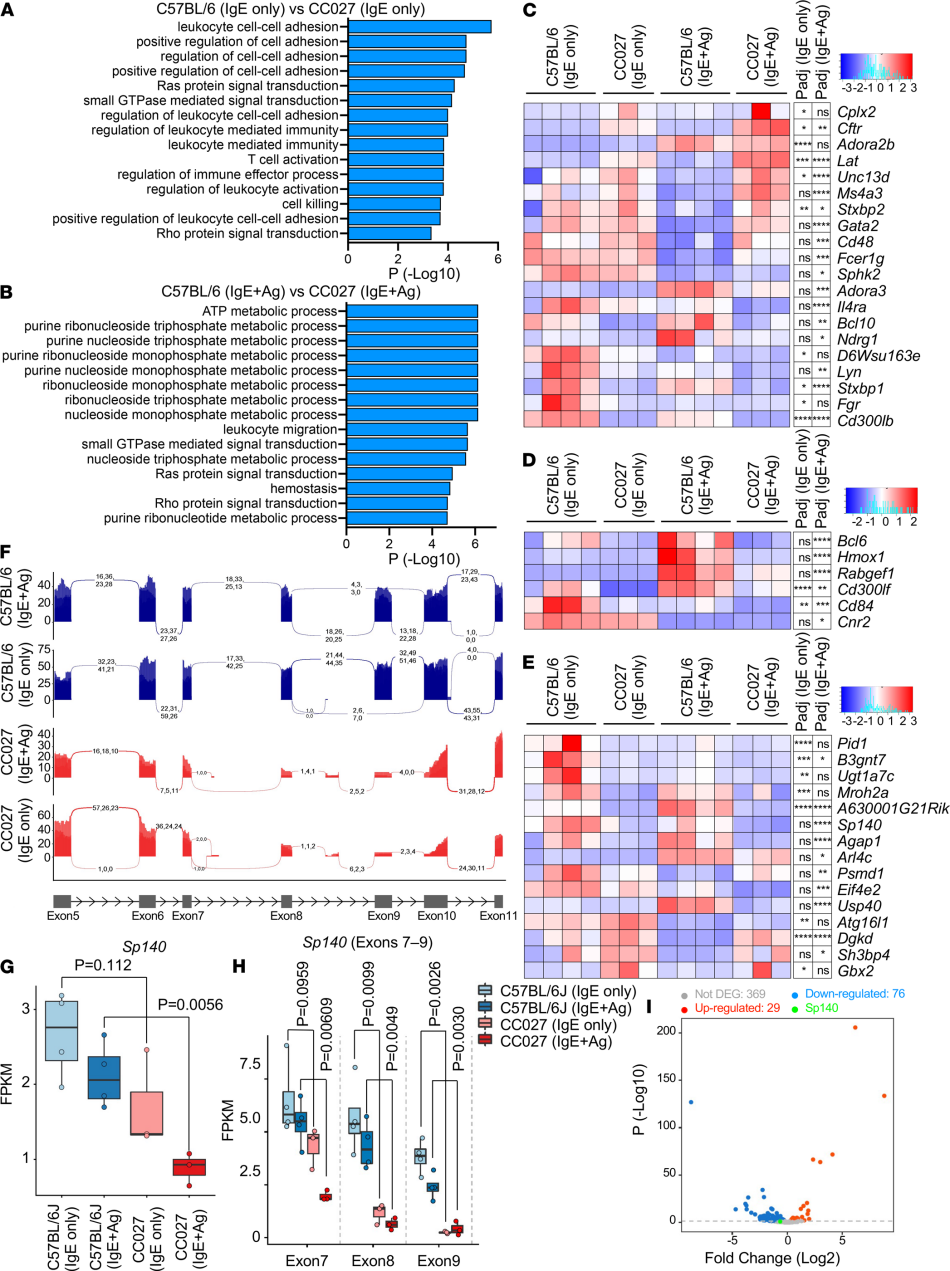
BMCMCs from CC027 and C57BL/6J mice have different RNA expression profiles. BM cells from CC027 and C57BL/6J mice were cultured with IL-3 for 6–7 weeks to generate BMCMCs. MCs were sensitized with mouse anti–DNP IgE mAb (1 μg/mL) overnight and then stimulated with (IgE+Ag) or without (IgE only) DNP-HSA (20 ng/mL) for 1 hour. The RNAs were collected and subjected to RNA sequencing. (**A** and **B**) GO biological processes significantly upregulated in CC027 BMCMCs stimulated without (**A**; IgE only) or with (**B**; IgE+Ag) DNP-HSA. (**C**–**E**) Heatmaps of genes in the GO category of positive (**C**) or negative (**D**) regulators of MC activation and of protein-coding genes in chromosome 1, region 81.7–92.9 Mb, or (**E**) differentially expressed between CC027 and C57BL/6J BMCMCs either with (IgE+Ag) or without (IgE only) DNP-HSA stimulation (*P <* 0.05). (**F**) Sashimi plots showing alternative splicing patterns for *Sp140* exons 5–10 in C57BL/6J (blue) and CC027 (red). (**G** and **H**) Expression levels (FPKM) of *Sp140* (**G**) and *Sp140* Exons 7–9 (**H**) in CC027 and C57BL/6J BMCMCs either with (IgE+Ag) or without (IgE only) DNP-HSA stimulation were extracted from RNA-seq results. (**I**) Volcano plot of *Sp140*-controlled genes (mouse macrophages) between unstimulated BMCMCs from CC027 or C57BL/6J mice. *n =* 3 for CC027 and *n =* 4 for C57BL/6J of different biological replicates. P_adj_: *P < 0.05, **P < 0.01, ***P < 0.001, ****P < 0.0001.

Gene ontology (GO) analysis revealed significant enrichment of the genes involved in several GTPase pathways in BMCMCs from CC027 mice (Figure 6, A and B, and Supplemental Table 5). GTPase-mediated signaling regulates several membrane trafficking events in cells, including IgE-dependent MC degranulation (42, 43). Therefore, the higher expression of GTPase pathway genes in CC027 MCs may contribute to their higher IgE-dependent degranulation. Additionally, BMCMCs from CC027 mice highly expressed some genes involved in MC activation, such as *Cftr*, *Lat*, *Unc13d*, *Ms4a3*, and *Stxbp2*, compared with the cells from C57BL/6J mice (Figure 6C and Supplemental Table 6A). On the other hand, the expression of negative regulators of MC activation — *Bcl6*, *Hmox1*, *Rabgef1*, *Cd300lf*, and *Cd84* — in CC027 strain–derived BMCMCs was lower than that in C57BL/6J strain–derived cells (Figure 6D and Supplemental Table 6B). This differential gene expression profile may directly contribute to the stronger MC activity in CC027 mice.

Because chromosome 1 at region 81.7–92.9 Mb was a potential QTL contributing to MC functions (Figure 4), we checked the differentially expressed genes in this region. Fifteen genes were identified as differentially expressed genes, and *Sp140* was on the list (Figure 6, E and G, and Supplemental Table 6C). We found 13 SNPs on *Sp140* mRNA expressed in CC027 BMCMCs, of which 10 were already reported, including 2 NOD/ShiLtJ strain–specific missense SNPs shown in the Sanger database (https://www.sanger.ac.uk) (Supplemental Table 7). In addition, we found 2 potentially novel missense SNPs and a synonymous SNP on CC027 *Sp140* mRNA. The sequencing results also showed lower abundances of exons 7–9 in CC027 *Sp140* mRNA, suggesting the presence of alternative mRNA transcripts skipping these exons (Figure 6, F and H, and Supplemental Figure 7A). This truncation would result in the expression of shorter *Sp140* transcripts devoid of a part of the SAND domain, a DNA-binding domain (Supplemental Figure 7, B and C) (44).

A previous study described *Sp140* as a regulator for TLR4-induced gene expression in mouse and human macrophages (34). Therefore, we compared previously reported *Sp140*-regulated genes in mouse macrophages (34) and genes differentially expressed between CC027 and C57BL/6J BMCMCs in our RNA-seq results. We found a significant overlap of differentially expressed genes between the 2 data sets (*P =* 3.608 × 10^–8^; Figure 6I and Supplemental Table 8A). Furthermore, we performed pathway analysis using the overlapping genes and found several interesting GO terms such as “regulation of immune effector process,” “T cell mediated immunity,” and “positive regulation of T cell mediated immunity” describing the upregulated genes (Supplemental Figure 8 and Supplemental Table 8B). These results suggest that the potential contribution of SNPs on *Sp140* to the differential gene expression profile between CC027 and C57BL/6J BMCMCs, and the effects of these *Sp140*-controlled genes, may at least partially account for the strong IgE-mediated responses in CC027 BMCMCs. Indeed, *Sp140* could be a novel regulator of MC function, and related immune responses, in vivo.

### Sp140 controls IgE-dependent activation of C57BL/6J BMCMCs.

**Figure 7:**
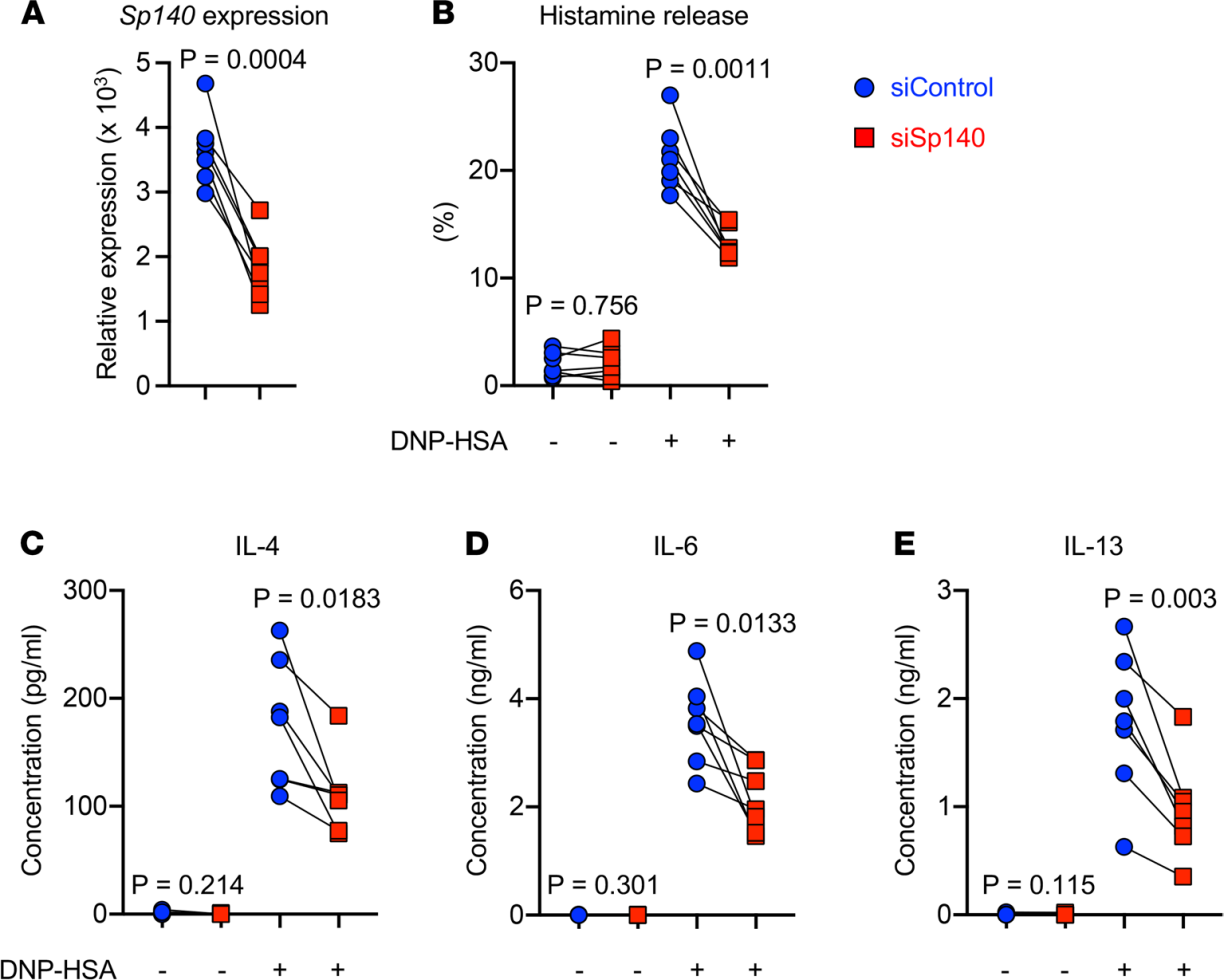
*Sp140* contributes to IgE-dependent MC responses. BM cells from C57BL/6J mice were cultured with IL-3 for 6–7 weeks to generate BMCMCs. Control siRNA or siRNA against *Sp140* were transfected into the cells. (**A**) mRNA level for *Sp140* 24 hours after the siRNA transfection was determined by quantitative PCR. The *Sp140* levels are indicated as relative to *Actb*. (**B**) siRNA-transfected cells were sensitized with mouse anti–DNP IgE mAb (1 μg/mL) overnight and then stimulated with DNP-HSA (10 ng/mL) for 1 hour. Histamine contents in cell lysates and the culture supernatants were determined by ELISA. Histamine release rates are indicated as percent release (supernatants) out of total histamine contents of the cells (cell lysate plus supernatant). (**C**–**E**) siRNA-transfected cells were stimulated as in **B** but for 6 hours. The levels of IL-4 (**C**), IL-6 (**D**), and IL-13 (**E**) in the culture supernatants were determined by ELISA. Each dot indicates different biological replicates. *n =* 7 pooled from 4 independent experiments. *P* values were determined by paired Student’s *t* test.

To examine the extent to which Sp140 is involved in IgE-dependent MC activation, we introduced an *Sp140*-specific siRNA into BMCMCs derived from C57BL/6J mice. Transfection of the *Sp140*-specific siRNA resulted in a 32%–58% reduction of *Sp140* mRNA levels compared with the control siRNA transfection (Figure 7A). *Sp140*–knocked-down MCs showed a significantly lower IgE ligation induced histamine release (~35% reduction) compared with the control cells (Figure 7B). Concomitantly, the production of IL-4, IL-6, and IL-13 also was significantly decreased in *Sp140*–knocked-down MCs (Figure 7, C–E).

Sp100 is in the same protein family with Sp140 (45) and is a candidate for MC activation, as NOD/ShiLtJ has a unique protein-changing SNP on the gene (Supplemental Table 4). However, neither histamine release nor cytokine production after IgE ligation was affected by the transfection of an *Sp100*-specific siRNA (Supplemental Figure 9). These results demonstrate that Sp140, but not Sp100, is essential for optimal MC degranulation and cytokine production in response to the tested level of IgE ligation.

## Discussion

Translational research increasingly requires the use of mouse models that accurately reflect human conditions (19, 20). However, mouse models employing traditional inbred strains cannot take into consideration the diverse genetic background of humans. CC mice can help to address this problem, as they cover approximately 90% of the genetic diversity of *Mus musculus* and they can be used under reproducible experimental conditions (21). Human genetic diversity influences many aspects of immunity, and the intensity of IgE-dependent human MC degranulation in vitro varies widely across MCs from different donors (46, 47). Here, we show that CC mouse strains exhibit marked variation in the intensity of their PCA reactions, a classical IgE-dependent MC response, in vivo. CC mice also exhibit substantial diversity in their resistance to *S.v.*, which significantly correlates with their PCA intensity. Therefore, it appears that CC mice represent good models for studies of the genetic diversity of IgE- and/or MC-dependent responses. Notably, we tested the same mice for both PCA reactivity and response to *S.v.* infection. Although all of the mice were rested for a week after the induction of the PCA reaction before transferring *S.v.*, we can’t formally rule out potential effects of their prior use for PCA reactions on the results of the subsequent infection with *S.v.*

Until now, the functions of mouse MCs have been studied primarily using mice on the C57BL/6J background (18). Our screening indicates that this strain is a reasonable model for studying IgE and MC functions in vivo because of the moderate strength of its IgE-dependent PCA reaction. Indeed, for C57BL/6J mice, all the parameters we screened were close to the average or median, and most of the parameters were within the interquartile range, of all the mice examined. Studying several different strains will help in developing the full picture of IgE- and MC-dependent biological responses, and our results were generated using a single classical model of such a response (i.e., the PCA reaction). However, our findings suggest that C57BL/6J mice may represent an average *Mus musculus* in expression of IgE- and MC-mediated immune responses.

By contrast, we found that BALB/cJ was an unusual strain, which showed an extremely low PCA reaction but a relatively quick expulsion of *S.v.* Notably, a lack of MCs delays *S.v.* expulsion more significantly on the C57BL/6J background than on the BALB/cJ background (5). Therefore, BALB/cJ mice may rely more on type-2 immune cells other than MCs, such as Th2 cells, to fight this parasite. The differences in Th2 immunity between C57BL/6J and BALB/cJ mice might also help to explain why a lack of MCs largely abrogated OVA-induced airway inflammation on the C57BL/6J background but not on the BALB/cJ background (12, 17). Indeed, this peculiarity of Th2 immunity in BALB/cJ mice needs to be considered when interpreting experimental results regarding the potential roles of MCs in this strain.

Among the parameters screened, PCA intensity, rapidity of *S.v.* expulsion, and fold change of serum IgE concentrations were significantly correlated. It is reasonable that resistance to *S.v.* correlated with the PCA, as both MCs and IgE are essential for normal immunity against this parasite (5). It is notable that PCA activity also correlated with IgE production. In a mouse model of food allergy, IgE-dependent MC activation, and IL-4 production from MCs, are essential for intestinal Th2 responses and allergen-specific IgE production (48). Moreover, mouse MCs have been shown to function as antigen-presenting cells (49, 50) or to stimulate B cell IgE production (51, 52) under certain circumstances in vitro. Taken together with our results, these findings suggest that further work on the potential contributions of MCs to the production of IgE in type-2 immunity may be of interest.

We found that some CC strains, including CC027, showed extremely potent PCA reactions. CC027 mice also exhibited the fastest *S.v.* expulsion, as well as the highest IgE fold change and the highest PBL eosinophil ratio, after *S.v.* infection. CC027 mice, thus, have a markedly strong type-2 response. Notably, Orgel et al. demonstrated that, among 16 CC stains they examined in addition to C57BL/6J, C3H/HeJ, and BALB/cJ mice, only CC027 mice showed hypothermia after an oral peanut allergen challenge in a food allergy model (26). They reasoned that the higher allergen absorption, probably caused by the higher intestinal allergen permeability of the strain, contributed to its anaphylaxis-prone phenotype.

In our study, we found that CC027 mice exhibited very high levels of IgE-dependent MC activity both in vivo and in vitro. The factors released from MCs, such as histamine, cytokines, and proteases, could compromise mucosal barrier function (53, 54). Indeed, MC number and/or activation correlates with enhanced intestinal permeability both in humans (55, 56) and in mice (57, 58). Therefore, the robust MC activity in strain CC027 may contribute to its higher allergen absorption in the intestine and its anaphylaxis-prone phenotype in the food allergy model. For these reasons, CC027 mice could be an attractive model for studying allergic and other MC-related diseases.

In contrast to the BM cells from all of the other mice tested, BM cells from CC012 mice did not produce MCs when placed in culture with IL-3. Interestingly, CC012 mice also showed the lowest jejunal and peritoneal MC numbers and serum Mcpt1 levels after *S.v.* infection. Because IL-3 plays a critical role in expanding MCs after *S.v.* infection (59), CC012 BM cells could have a defect in and/or downstream of IL-3 receptor signaling. These observations provide additional evidence that CC mice are an attractive tool for investigating the diversity of factors potentially influencing MC development both in the steady-state and under inflammatory conditions.

In our study, we identified *Sp140* as a potentially novel gene involved in IgE-dependent MC activation. Our QTL analysis showed that chromosome 1 in the interval of 81.7–92.9 Mb from NOD/ShiLtJ mice potentially contributed to a high PCA value, rapid *S.v.* expulsion, and high IgE fold change. Among the 100 protein-coding genes within the region, 4 had NOD/ShiLtJ-specific, protein-changing SNPs. *Col4a4* and *Col4a3* encode components of type IV collagen. While collagens could affect the MCs’ tissue interactions, it seems less likely that collagens control cell-intrinsic MC functions.

By contrast, *Sp140* and *Sp100* belong to the same nuclear protein family that has multiple DNA-binding domains (45). SNPs on *SP140* associate with Crohn’s disease (34, 35) and multiple sclerosis (37) in humans. Moreover, a GO analysis of genes whose expression positively correlated with *SP140* expression in intestinal biopsies revealed MC activation as one of the strong hit gene signatures (34). In addition, in vitro siRNA-mediated knock-down of *Sp140* in mouse macrophages demonstrated that the gene contributes to the LPS-induced proinflammatory response (34).

Our RNA-seq results confirmed that 2 NOD/ShiLtJ-specific missense SNPs on *Sp140* mRNA were expressed in CC027 BMCMCs and also revealed previously unknown missense SNPs and mRNA transcripts (skipping exons 7–9). Crohn’s disease–associated SNPs on *SP140* alter the mRNA splicing and skip its exon 7 in humans (34, 36). Also, we showed a similarity between published *Sp140*-controlled genes in mouse macrophages (34) and the differentially expressed genes between C57BL/6J and CC027 BMCMCs in our study.

These observations strongly suggested that NOD/ShiLtJ-specific SNPs on *Sp140* contributed to the strong PCA reactions, rapid *S.v.* expulsion, and marked changes in IgE observed in CC mice. Indeed, we showed that siRNA-mediated knock-down of *Sp140,* but not of *Sp100*, in C57BL/6J BMCMCs resulted in the decreased IgE-dependent MC release of histamine and IL-4, IL-6, and IL-13. While such results show that *Sp140* is essential for these important aspects of MC activation in C57BL/6J mice, it will be of interest to determine the effects of *Sp140* on the release of these and additional MC cytokines, and other mediators, in diverse mice.

For example, although CC027 BMCMCs, which have higher MC activities than C57BL/6J BMCMCs, showed lower *Sp140* expression levels, knockdown of the gene in C57BL/6J BMCMCs resulted in decreased MC activity. Therefore, we suspect that the differential Sp140 protein structure caused by the SNPs or the truncation, rather than the expression level itself, may modify Sp140’s function and contribute to the higher MC activity in CC027 mice. However, further studies are needed to uncover the exact role of Sp140 in MC activation and how these SNPs confer stronger MC activity in the CC027 strain.

Although we found *Sp140* to be a potential regulator of MC function in CC027 mice, we cannot rule out the potential involvement of immune cells other than MCs in CC027’s high resistance against *S.v.* infection. Indeed, *Sp140* controls macrophage function (34), and a subpopulation of macrophages, such as M2 macrophages, contributes to immunity against parasites (60, 61). Therefore, macrophages represent another candidate cell type that might contribute CC027’s high resistance to *S.v.* infection.

We also identified chromosome 10 in the interval of 79.4–94.0 Mb from the NZO/HILtJ strain as a potential QTL that negatively affects PCA, *S.v.* expulsion, and IgE fold change. Within that region, we found 4 genes — *Zbtb7a*, *Polr3b*, *Cfap54*, and *Usp44* — that had NZO/HILtJ strain–specific, protein-changing SNPs. *Zbtb7a* is a zinc finger/BTB domain–containing transcription factor with multiple functions in cell proliferation and hematopoiesis (62) whose expression in MCs is reportedly high (63). *Polr3b* encodes a subunit of RNA polymerase III, which regulates innate immune responses such as intracellular DNA sensing (64, 65) and TLR signaling pathways (66, 67). Therefore, these genes could be attractive candidates for modifying MC activation.

In conclusion, we have demonstrated that (a) there is a diverse range of IgE-dependent MC activation and MC-related immune responses in CC mice, (b) the C57BL/6J strain is a reasonable model for studying IgE-dependent MC functions in mice in vivo, (c) CC027 is a mouse strain that shows notably strong MC-dependent and type-2 immune responses, and (d) *Sp140* is a potentially novel gene contributing to IgE-dependent MC activation in mice. We suggest that CC mice could be useful for studying disease models where the role of MCs has not been clearly demonstrated using traditional inbred mouse strains. Indeed, CC mice might help to uncover a broader spectrum of MC functions in health and disease.

## Methods

### Study design.

For screening using CC mice, we prepared 3 or 5 mice per genotype. Some mice died before the beginning of the screening or after we performed PCA. Therefore, *n =* 3 or 5 for most of the data, but some are *n =* 2 or 1 (see detailed methods for screening protocol using CC mice and Supplemental Table 1). The screening was performed as 8 independent experiments, and the data were pooled. To validate the accuracy of the screening, we performed a second round of screening using a small number of strains (4 CC strains plus C57BL/6J and BALB/cJ mice). For this, we prepared *n =* 4 for CC027 and *n =* 6 for other strains. The second-round screening was performed as 3 independent experiments, and the data were pooled. For in vitro experiments with BMCMCs, we prepared more than *n =* 3 of biological replicates (MCs from different mice). RNA-seq was performed as a single experiment, and other in vitro experimental data were pooled from more than 2 independent experiments. No sample was excluded. Randomization and blinding were not used in the experiments.

### Experimental animals.

Female CC mice were purchased from the University of North Carolina (UNC) Systems Genetics Core. Female C57BL/6J and BALB/cJ mice were purchased from The Jackson Laboratory or Oriental Yeast. BALB/cJ *Rag1*^–/–^ *Il2rg*^–/–^ mice (68) were provided by Irving Weissman (Stanford University School of Medicine). Wistar rats were purchased from Charles River Laboratories. Mice and rats were maintained under specific pathogen–free conditions at the animal facilities in Stanford University or Hyogo College of Medicine.

### Antibodies and reagents.

Anti–DNP IgE mouse mAb (clone ɛ26) (69) was provided by Fu-Tong Liu (UC Davis Department of Dermatology, Davis, California, USA). mAbs specific for mouse B220 (clone RA3-6B2) Pacific blue–conjugated, CD3ε (clone 17A2) Pacific blue–conjugated, CD11c (N418) FITC–conjugated, CD16/32 (clone 93) purified, CD49b (clone DX5) PerCP-Cy5.5–conjugated, CD117 (clone 2B8) PE–conjugated, and Ly-6G/Ly-6C (clone Gr-1; RB6-8C5) Pacific blue–conjugated purified mouse IgE k isotype control, as well as purified mouse IgG1 k isotype control, were from BioLegend. mAbs specific for mouse IgE (clone R35-72) FITC–conjugated, CD11b (clone M1/70) PE–conjugated, Siglec-F (clone E50-2440) PerCP-Cy5.5–conjugated, IgE (clone R35-72) purified, IgE (clone R35-72) Biotin–conjugated, IgG1 (clone A85-3) purified, and IgG1 (clone A85-1) Biotin–conjugated antibodies, as well as Streptavidin-HRP, were from BD Biosciences. Recombinant Murine IL-3 was from PeproTech. Albumin DNP-HSA; 3, 3′, 5, 5′-Tetramethylbenzidine Liquid Substrate, Supersensitive, for ELISA; Minimum Essential Medium Eagle; PIPES; and Tyrode’s Salt were from Sigma-Aldrich.

### Screening protocol using CC mice.

The screening protocol is depicted in Figure 1A. Sera from naive mice were obtained 1 week before the beginning of the PCA (day –7). Mice then were passively sensitized with IgE by intradermal injection of mouse anti–DNP IgE (20 ng/20 μL/ear) in the right ear pinna (day 0). The same volume of vehicle (HMEM/Pipes buffer; MEM with HBBS containing 0.47 g/L pipes) was injected into left ear pinna as a control. Twenty-four hours later (day 1), the mice were i.v. injected with DNP-HSA (100 μg/100 μL/dose) using the Extreme Microcyringe (Ito Corporation). Ear thickness was measured immediately before (0 hour) and 0.5, 1, 2, 4, 6, and 8 hours after antigen challenge using a dial thickness gauge (Ozaki MFG). The increase in ear thickness (Δ ear thickness) was calculated by subtracting the values of 0 hour from each time point of induced (right) minus control (left) ear thickness. On day 8 (7 days after PCA), mice were infected with *S.v.* by s.c. injection of 5000 L3 larvae. The number of *S.v.* eggs in the stool was counted every day microscopically from day 6 after infection (day 13) until the mice stopped producing the eggs. The date from the beginning of infection to the date the mice stopped producing *S.v.* eggs was considered as the duration of *S.v.* infection. Three days after the mice stopped producing *S.v.* eggs, mice were sacrificed and the animals’ peritoneal cells and blood samples were analyzed by FACS and ELISA. Quantities include *n =* 2 for CC007, CC030, CC032, CC038, CC040, CC051, and CC060; *n =* 5 for CC015, CC029, CC053, C57BL/6J, and BALB/cJ; and *n =* 3 for rest of the strains. After measuring the PCA reactions, 1 CC019, 1 CC053, and 2 CC012 mice died; therefore, quantities include *n =* 2 for CC019, *n =* 4 for CC053, and *n =* 1 for CC012 after *S.v.* infection (Supplemental Table 1).

### QTL analysis.

QTL mapping was performed on the phenotypes of maximum PCA value, *S.v.* expulsion date, and IgE fold change across CC mice strains. We used DOQTL v1.19.0 Bioconductor package (70) for QTL mapping. Genotype markers and haplotype probabilities for the CC mice were obtained from the Systems Genetics Core Facility at the UNC (http://csbio.unc.edu/CCstatus/index.py). The log odds (LOD) score was calculated, comparing the observations according to phenotype with and without the founder genotype probabilities at each locus (70). We determined the statistical significance of the LOD score with 1000 permutations.

### Preparation and maintenance of S.v.

*S.v.* was maintained in female Wistar rats or BALB/cJ *Rag1*^–/–^ *Il2rg*^–/–^ mice as previously described (5, 59).

### Histological examination.

Mouse ears, back skins, and jejunums were fixed with 10% formalin and were paraffin-embedded. Then, 4 μm sections were prepared. For ears and back skins, the samples were stained with 0.1% Toluidine blue. Jejunum samples were incubated in 0.5% Toluidine blue for 48 hours at room temperature. The numbers of MCs in the tissues were quantified by microscopy.

### FACS analysis.

Peritoneal cells were collected from mice by injecting PBS (5 mL, 4°C) into the peritoneum. Blood samples were obtained using heparin-coated capillary tubes centrifuged at 4°C (2000*g*, 5 minutes), and the cell pellets were suspended with Gibco ACK lysing buffer (Thermo Fisher Scientific) to obtain peripheral blood cells. Cells were washed in ice-cold staining buffer (1% BSA-PBS), Fc-blocked with anti-CD16/32 antibody; they were then incubated with antibodies against CD49b, CD117, IgE, and CD3/B220/Gr-1 for peritoneal cells and CD11b, CD11c, Gr-1, and Siglec-F for peripheral blood cells (all 1/100) for 15 minutes at 4°C and washed twice with staining buffer. Data were acquired using a FACS Canto II flow cytometer (BD Biosciences) and analyzed using FlowJo software (version 7.6.1, Tree Star Inc.). Peritoneal MCs were defined as CD3^–^B220^–^Gr-1^–^CD49b^–^CD117^+^IgE^+^ cells, and peripheral blood eosinophils were defined as CD11b^+^Gr-1^–^Siglec-F^+^ cells.

### Serum Ig levels.

Levels of mouse serum IgG1 and IgE were measured by ELISA using anti–mouse IgG1 mAb (clone A85-3) or anti–mouse IgE mAb (clone R35-72) as capture antibodies, anti–mouse IgG1 mAb biotin–conjugated (clone A85-1) or anti–mouse IgE mAb biotin–conjugated (clone R35-118) as secondary antibodies, and Streptavidin-HRP (BD Biosciences) and 3, 3′, 5, 5′-Tetramethylbenzidine Liquid Substrate, Supersensitive, for ELISA (Sigma-Aldrich), as detection reagents. Purified mouse IgG1 κ isotype control and purified mouse IgE κ isotype control (BioLegend) were used as ELISA standards.

### Preparation of BMCMCs.

BMCMCs were generated from BM cells derived from CC, C57BL/6J, or BALB/cJ mice. BM cells were cultured in DMEM containing 10% FBS and 10 ng/mL mouse IL-3 (at 37°C) for 6–7 weeks. To induce IgE-dependent MC responses, BMCMCs were sensitized with anti–DNP IgE (1 μg/mL) overnight at 37°C; then, they were stimulated with DNP-HSA (20 ng/mL) in Tyrode’s buffer (Tyrode’s salt solution containing 10 mM HEPES, 1% glucose, and 1% BSA) for 1 hour at 37°C (to examine histamine release) or in DMEM culture medium for 24 hours at 37°C (to examine cytokine production). To examine MC histamine release and cytokine production, cells were incubated using U-bottomed (for histamine release) or flat-bottomed (for cytokine production) 96-well plates (1.5 × 10^5^ MCs/200 μL/well). In some experiments (for siRNA-mediated gene knockdown), we stimulated the cells with DNP-HSA (10 ng/mL) for 6 hours for cytokine production. Because we assumed that gene silencing could result in a higher MCs response, we decided to use a suboptimal dose of DNP-HSA (10 ng/mL). Also, because we noticed that the knockdown effect did not last very long, we used 6 hours of MC stimulation to shorten the time after the siRNA transfection.

### RNA-seq.

BMCMCs were generated from BM cells derived from CC027 and C57BL/6J mice. Cells were sensitized with anti–DNP IgE (1 μg/mL) overnight at 37°C; they were then stimulated with or without DNP-HSA (20 ng/mL) for 1 hour at 37°C. Total RNAs were extracted from BMCMCs using RNeasy Micro kit (Qiagen). RNA-seq library construction and sequencing was done by Novogene (https://en.novogene.com/). Paired-end 150-bp sequencing was performed on Illumina HiSeq 2000 instruments. Fastq files from all samples were aligned via STAR v2.7.1a (71) using a mm10 genome reference and GENCODE vM21 annotations. To quantify the abundance of each gene and isoform, we used RSEM v1.3.1 (72) to calculate fragments per kilobase of transcript per million mapped reads (FPKM) based on the length and the read count mapped to each gene or isoform. Differential expression analysis of C57BL/6J and CC027 was performed using the DESeq2 R package (1.24.0; ref. 73). Differentially expressed genes were determined at an adjusted *P* value threshold of 0.05. GO enrichment analysis of those genes was performed using clusterProfiler R package (3.12.0; ref. 74). RNA-seq raw files are available through the NCBI GEO database (accession no. GSE174030).

### siRNA transfection.

Ambion Silencer Select Predesigned siRNA against *Sp140* (s204308) or *Sp100* (s74199), or Ambion Silencer Select Negative Control siRNA (Thermo Fisher Scientific), were transfected into BMCMCs using Amaxa Mouse Macrophage Nucleofector Kit and Nucleofector II device (Lonza). In total, 2 × 10^6^ BMCMCs were transfected with 500 nM siRNAs at 37°C. Six hours after the transfection, cells were sensitized with anti–DNP IgE (1 μg/mL) at 37°C, and 24 hours after the transfection, cells were stimulated with DNP-HSA (10 ng/mL) for 1 or 6 hours at 37°C to determine histamine release and cytokine production, respectively. The mRNA levels for *Sp140* or *Sp100* were determined 24 hours after the siRNA transfection at 37°C.

### Cytokine and histamine ELISA.

Cytokine concentrations in culture supernatants were analyzed by mouse ELISA DuoSet ELISA kits for IL-4, IL-6, and IL-13 (R&D Systems); mouse IL-4 and IL-13 ELISA Ready Set Go kits (eBioscience) or Mouse IL-6 ELISA Kit (Invitrogen). Histamine concentrations in culture supernatants were analyzed by EIA Histamine (Beckman Coulter).

### Quantitative PCR (qPCR).

Total RNAs from BMCMCs were isolated using the RNeasy Mini Kit (Qiagen). cDNA was synthesized using High Capacity cDNA Reverse Transcription Kit (Thermo Fisher Scientific) or ReverTra Ace qPCR RT Master Mix (Toyobo). Gene expression levels were quantified using TaqMan Gene Expression Assays (Thermo Fisher Scientific), Premix Ex Taq (Perfect Real Time), and the Thermal cycler dice real-time PCR system (Takara Bio Inc.), with an initial denature at 95°C for 30 s, followed by 40 to 45 cycles of denaturing at 95°C for 5 seconds and annealing and extension at 60°C for 30 seconds. The TaqMan probes used in this study were Applied Biosystems *Sp140* (Mm00439646_m1), *Sp100* (Mm00434305_m1), and mouse *Actb* (Thermo Fisher Scientific). Results are shown relative to *Actb* levels as determined by the 2^–ΔΔCt^ method.

### Statistics.

Two-tailed Student’s *t* test (to compare 2 groups), 1-way ANOVA followed by Tukey’s test (to compare more than 2 groups), and Spearman’s rank correlation coefficient were performed using Prism 6 (version 6.0h, GraphPad Software). *P* values less than 0.05 were considered statistically significant.

### Study approval.

The use of all mice for these studies was in accord with institutional guidelines, with review and approval by the Stanford IACUC (no. 12683) or Institutional Animal Care Committee of Hyogo College of Medicine (no. 19–015).

## Author contributions

K. Matsushita, MT, and SJG designed the study; K. Matsushita, YN, and K. Mukai performed experiments; XL, DD, and SBM performed computational analysis; K. Matsushita, XL, DD, SBM, MT, and SJG analyzed data and wrote the manuscript; and SBM and SJG supervised the study. K. Matsushita is designated the first co–first author because he performed most of the in vivo and in vitro experiments and completed the study. XL is designated the second co–first author because he performed the extensive computational analysis of the data obtained from the experiments. All authors discussed the results and revised the manuscript.

## Supplementary Material

Supplemental data

Supplemental Table 1

Supplemental Table 2

Supplemental Table 3

Supplemental Table 4

Supplemental Table 5

Supplemental Table 6

Supplemental Table 7

Supplemental Table 8

## Version 1 06/22/2021

Electronic publication

## References

[1] Olivera A (2018). Mast cells signal their importance in health and disease. J Allergy Clin Immunol.

[2] Wernersson S, Pejler G (2014). Mast cell secretory granules: armed for battle. Nat Rev Immunol.

[3] Galli SJ (2020). Mast cells and IgE in defense against lethality of venoms: Possible “benefit” of allergy[]. Allergo J Int.

[4] Starkl P (2020). IgE effector mechanisms, in concert with mast cells, contribute to acquired host defense against staphylococcus aureus. Immunity.

[5] Mukai K (2017). Differences in the importance of mast cells, Basophils, IgE, and IgG versus that of CD4^+^ T cells and ILC2 cells in primary and secondary immunity to strongyloides venezuelensis. Infect Immun.

[6] Galli SJ, Tsai M (2012). IgE and mast cells in allergic disease. Nat Med.

[7] Cildir G (2019). Genome-wide analyses of chromatin state in human mast cells reveal molecular drivers and mediators of allergic and inflammatory diseases. Immunity.

[8] Brown MA, Hatfield JK (2012). Mast cells are important modifiers of autoimmune disease: with so much evidence, why is there still controversy?. Front Immunol.

[9] Heijmans J (2012). Role of mast cells in colorectal cancer development, the jury is still out. Biochim Biophys Acta.

[10] Marichal T (2013). Mast cells: potential positive and negative roles in tumor biology. Cancer Immunol Res.

[11] Yamashita Y (2007). Cutting edge: genetic variation influences Fc epsilonRI-induced mast cell activation and allergic responses. J Immunol.

[12] Becker M (2011). Genetic variation determines mast cell functions in experimental asthma. J Immunol.

[13] Fernando J (2013). Genotype-dependent effects of TGF-beta1 on mast cell function: targeting the Stat5 pathway. J Immunol.

[14] Beghdadi W (2011). Mast cells as cellular sensors in inflammation and immunity. Front Immunol.

[15] Schubert N (2015). Mast cell promotion of T cell-driven antigen-induced arthritis despite being dispensable for antibody-induced arthritis in which T cells are bypassed. Arthritis Rheumatol.

[16] Schubert N (2018). Unimpaired responses to vaccination with protein antigen plus adjuvant in mice with kit-independent mast cell deficiency. Front Immunol.

[17] Hernandez JD (2020). Development of multiple features of antigen-induced asthma pathology in a new strain of mast cell deficient BALB/c-Kit(W-sh/W-sh) mice. Lab Invest.

[18] Galli SJ (2020). Mast cells in inflammation and disease: recent progress and ongoing concerns. Annu Rev Immunol.

[19] Masopust D (2017). Of mice, dirty mice, and men: using mice to understand human immunology. J Immunol.

[20] Hamilton SE (2020). New insights into the immune system using dirty mice. J Immunol.

[21] Churchill GA (2004). The Collaborative Cross, a community resource for the genetic analysis of complex traits. Nat Genet.

[22] Threadgill DW, Churchill GA (2012). Ten years of the Collaborative Cross. Genetics.

[23] Graham JB (2017). Extensive homeostatic T cell phenotypic variation within the Collaborative Cross. Cell Rep.

[24] Legrand JMD (2018). Genetic variation in the mitogen-activated protein kinase/extracellular signal-regulated kinase pathway affects contact hypersensitivity responses. J Allergy Clin Immunol.

[25] Rasmussen AL (2014). Host genetic diversity enables Ebola hemorrhagic fever pathogenesis and resistance. Science.

[26] Orgel K (2019). Genetic diversity between mouse strains allows identification of the CC027/GeniUnc strain as an orally reactive model of peanut allergy. J Allergy Clin Immunol.

[27] Rogala AR (2014). The Collaborative Cross as a resource for modeling human disease: CC011/Unc, a new mouse model for spontaneous colitis. Mamm Genome.

[28] Wershil BK (1987). 125I-fibrin deposition in IgE-dependent immediate hypersensitivity reactions in mouse skin. Demonstration of the role of mast cells using genetically mast cell-deficient mice locally reconstituted with cultured mast cells. J Immunol.

[29] Okudaira H (1980). A study of the rat passive cutaneous anaphylaxis (PCA) reaction for the assay of mouse IgE antibody. J Immunol Methods.

[30] Nguyen M (2005). Age-induced reprogramming of mast cell degranulation. J Immunol.

[31] Heinzel FP (1989). Reciprocal expression of interferon gamma or interleukin 4 during the resolution or progression of murine leishmaniasis. Evidence for expansion of distinct helper T cell subsets. J Exp Med.

[32] Keane TM (2011). Mouse genomic variation and its effect on phenotypes and gene regulation. Nature.

[33] Yalcin B (2011). Sequence-based characterization of structural variation in the mouse genome. Nature.

[34] Mehta S (2017). Maintenance of macrophage transcriptional programs and intestinal homeostasis by epigenetic reader SP140. Sci Immunol.

[35] Jostins L (2012). Host-microbe interactions have shaped the genetic architecture of inflammatory bowel disease. Nature.

[36] Matesanz F (2015). A functional variant that affects exon-skipping and protein expression of SP140 as genetic mechanism predisposing to multiple sclerosis. Hum Mol Genet.

[37] International Multiple Sclerosis Genetics C (2013). Analysis of immune-related loci identifies 48 new susceptibility variants for multiple sclerosis. Nat Genet.

[38] Ibrahim MZ (1996). The mast cells of the multiple sclerosis brain. J Neuroimmunol.

[39] Kallweit U (2013). Elevated CSF histamine levels in multiple sclerosis patients. Fluids Barriers CNS.

[40] Araki Y (1993). Mast cells and histamine release in Crohn’s disease. Kurume Med J.

[41] Gelbmann CM (1999). Strictures in Crohn’s disease are characterised by an accumulation of mast cells colocalised with laminin but not with fibronectin or vitronectin. Gut.

[42] Prashar A (2017). Rab GTPases in immunity and inflammation. Front Cell Infect Microbiol.

[43] Draber P (2012). Cytoskeleton in mast cell signaling. Front Immunol.

[44] Bottomley MJ (2001). The SAND domain structure defines a novel DNA-binding fold in transcriptional regulation. Nat Struct Biol.

[45] Saare M (2015). SP140L, an evolutionarily recent member of the SP100 family, is an autoantigen in primary biliary cirrhosis. J Immunol Res.

[46] Babina M (2016). Phenotypic variability in human skin mast cells. Exp Dermatol.

[47] Babina M (2017). Skin mast cell phenotypes between two highly divergent cohorts - more pronounced variability within than between groups. Exp Dermatol.

[48] Burton OT (2014). Immunoglobulin E signal inhibition during allergen ingestion leads to reversal of established food allergy and induction of regulatory T cells. Immunity.

[49] Gaudenzio N (2009). Cell-cell cooperation at the T helper cell/mast cell immunological synapse. Blood.

[50] Nakano N (2009). Notch signaling confers antigen-presenting cell functions on mast cells. J Allergy Clin Immunol.

[51] Gauchat JF (1993). Induction of human IgE synthesis in B cells by mast cells and basophils. Nature.

[52] Ryzhov S (2004). Adenosine-activated mast cells induce IgE synthesis by B lymphocytes: an A2B-mediated process involving Th2 cytokines IL-4 and IL-13 with implications for asthma. J Immunol.

[53] Madden KB (2002). Role of STAT6 and mast cells in IL-4- and IL-13-induced alterations in murine intestinal epithelial cell function. J Immunol.

[54] Groschwitz KR (2009). Mast cells regulate homeostatic intestinal epithelial migration and barrier function by a chymase/Mcpt4-dependent mechanism. Proc Natl Acad Sci U S A.

[55] Cherner JA (1988). Gastrointestinal dysfunction in systemic mastocytosis. A prospective study. Gastroenterology.

[56] Lee H (2013). Mucosal mast cell count is associated with intestinal permeability in patients with diarrhea predominant irritable bowel syndrome. J Neurogastroenterol Motil.

[57] Ahrens R (2012). Intestinal mast cell levels control severity of oral antigen-induced anaphylaxis in mice. Am J Pathol.

[58] Leyva-Castillo JM (2019). Mechanical skin injury promotes food anaphylaxis by driving intestinal mast cell expansion. Immunity.

[59] Lantz CS (1998). Role for interleukin-3 in mast-cell and basophil development and in immunity to parasites. Nature.

[60] Ishii M (2009). Epigenetic regulation of the alternatively activated macrophage phenotype. Blood.

[61] Satoh T (2010). The Jmjd3-Irf4 axis regulates M2 macrophage polarization and host responses against helminth infection. Nat Immunol.

[62] Lunardi A (2013). Role of LRF/Pokemon in lineage fate decisions. Blood.

[63] Norton LJ (2017). KLF1 directly activates expression of the novel fetal globin repressor *ZBTB7A/LRF* in erythroid cells. Blood Adv.

[64] Chiu YH (2009). RNA polymerase III detects cytosolic DNA and induces type I interferons through the RIG-I pathway. Cell.

[65] Ablasser A (2009). RIG-I-dependent sensing of poly(dA:dT) through the induction of an RNA polymerase III-transcribed RNA intermediate. Nat Immunol.

[66] Graczyk D (2015). Involvement of RNA polymerase III in immune responses. Mol Cell Biol.

[67] Reverendo M (2019). Polymerase III transcription is necessary for T cell priming by dendritic cells. Proc Natl Acad Sci U S A.

[68] Cao X (1995). Defective lymphoid development in mice lacking expression of the common cytokine receptor gamma chain. Immunity.

[69] Liu FT (1980). Monoclonal dinitrophenyl-specific murine IgE antibody: preparation, isolation, and characterization. J Immunol.

[70] Gatti DM (2014). Quantitative trait locus mapping methods for diversity outbred mice. G3 (Bethesda).

[71] Dobin A (2013). STAR: ultrafast universal RNA-seq aligner. Bioinformatics.

[72] Li B, Dewey CN (2011). RSEM: accurate transcript quantification from RNA-Seq data with or without a reference genome. BMC Bioinformatics.

[73] Love MI (2014). Moderated estimation of fold change and dispersion for RNA-seq data with DESeq2. Genome Biol.

[74] Yu G (2012). clusterProfiler: an R package for comparing biological themes among gene clusters. OMICS.

