# Supplementary material for: The role of Sp140 revealed in IgE and mast cell responses in Collaborative Cross mice
Source: JCI Insight. 2021 Jun 22;6(12):e146572. doi: 10.1172/jci.insight.146572 (PMC8262499; doi:10.1172/jci.insight.146572)
Supplement: Supplemental data [file jciinsight-6-146572-s116.pdf]

IgE and mast cell responses in collaborative cross mice: Role of *Sp140*

Authors

Kazufumi Matsushita, Xin Li, Yuki Nakamura, Danyue Dong, Kaori Mukai, Mindy Tsai, Stephen B. Montgomery, Stephen J. Galli.

Supplemental Data

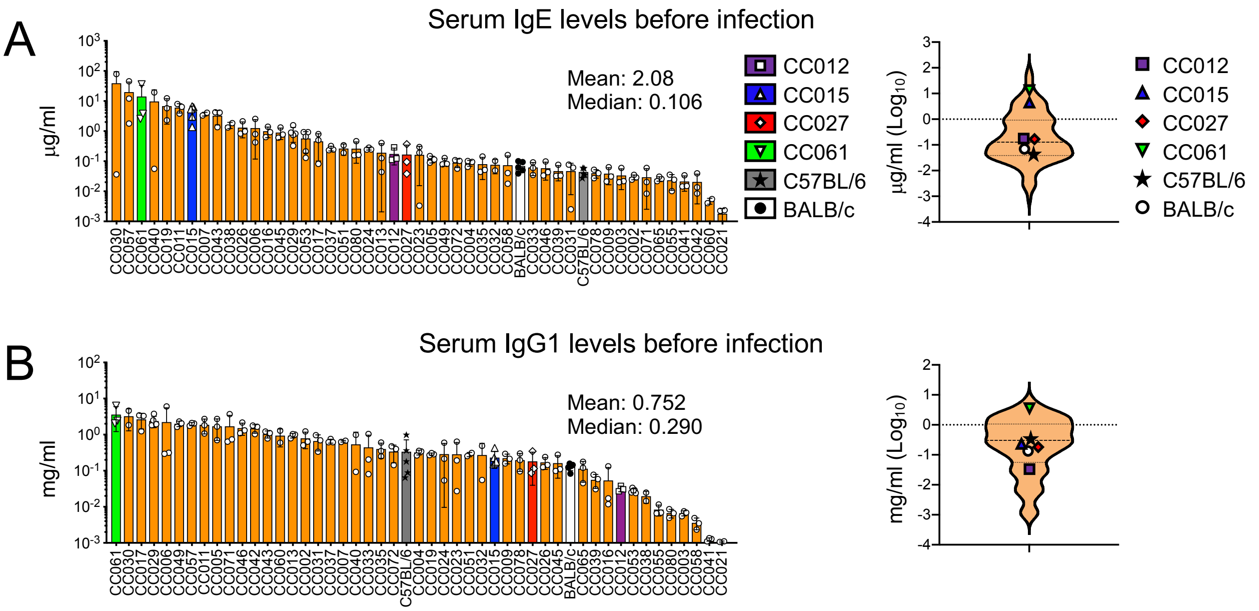

Supplemental Figure 1. Baseline levels of IgE and IgG1 in CC, C57BL/6J and BALB/c mice.
Serum samples were obtained from mice a week before the beginning of PCA (**Figure 1**). Summary graphs (left) and violin plots (right) of baseline levels of serum IgE (**A**) and IgG1 (**B**) for each mouse genotype are shown. n=1–5 mice per genotype (see **Supplemental Table 1**) from eight independent experiments. Purple, blue, red, green, black, and white lines, bars and dots represent CC012, CC015, CC027, CC061, C57BL/6J and BALB/c mice, respectively. Barplots with error bars for summary graphs indicate means ± SDs, and each dot indicates one mouse. Dashed lines in violin plots indicate 25th and 75th quartiles and median.

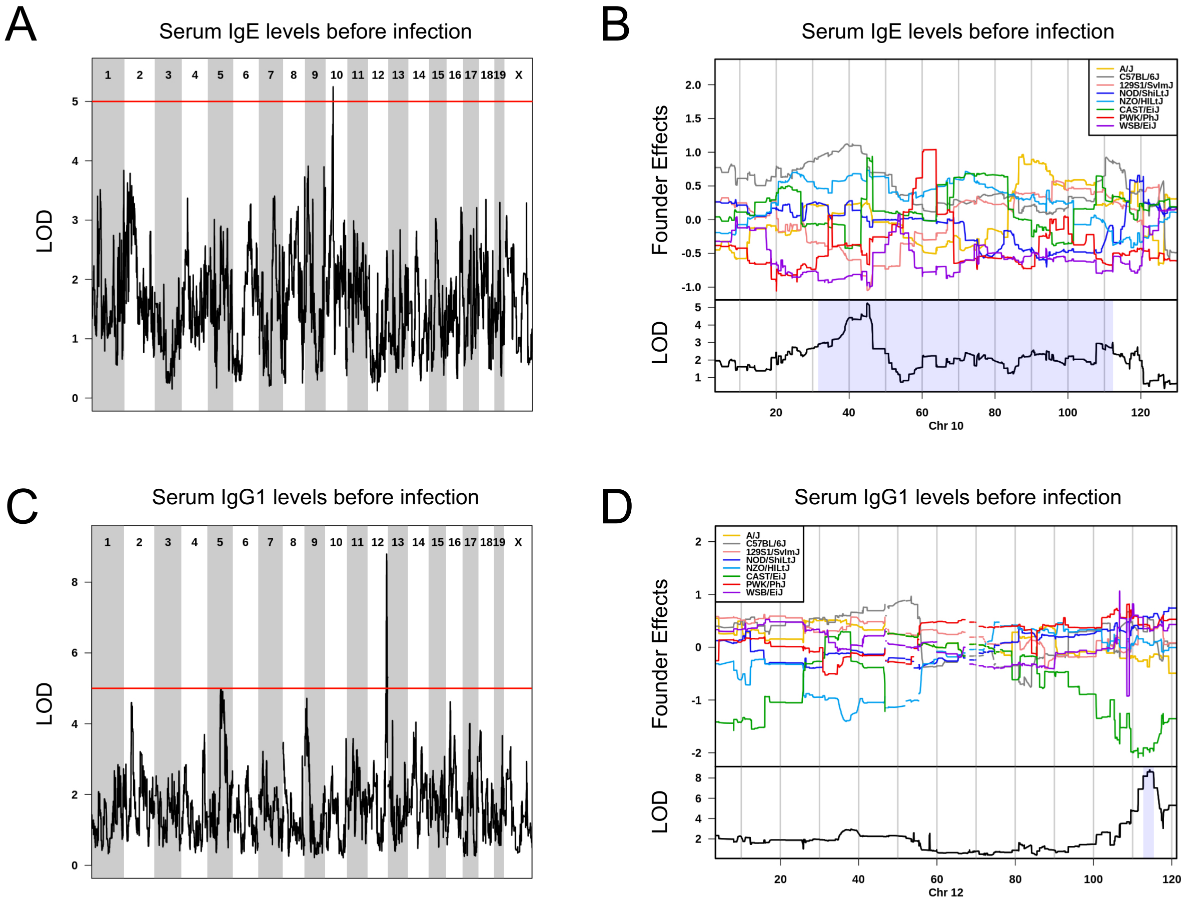

Supplemental Figure 2. QTL analysis for baseline levels of mouse IgE and IgG1.
(**A and C**) QTL analysis for chromosomal regions associated with baseline serum levels of IgE (**A**) and IgG1 (**C**). Red lines indicate the LOD score threshold for P = 0.05. (**B and D**) Analysis for founder effects associated with baseline serum IgE levels within chromosome 10 (**B**) and with baseline serum IgG1 levels within chromosome 12 (**D**).

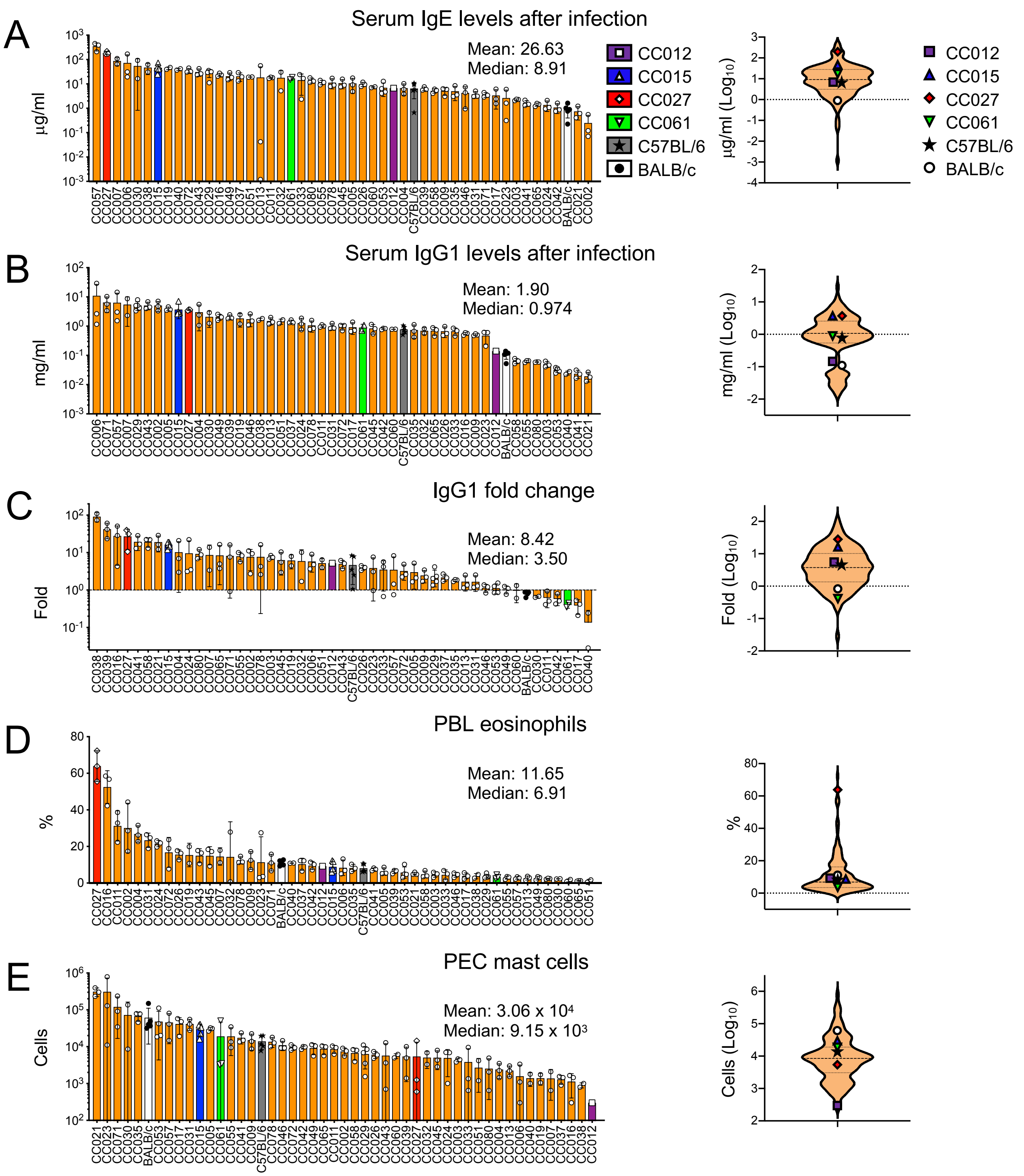

Supplemental Figure 3. Immunological parameters were assessed after *S. v.* infection in CC, C57BL/6J and BALB/c mice.

Mice were treated as in **Figure 1A**. All samples were obtained three days after the mice stopped producing *S. v.* eggs. Summary graphs (left) and violin plots (right) of serum IgE levels after *S. v.*-infection (**A**), serum IgG1 levels after *S. v.*-infection (**B**) and the IgG1 fold change from the baseline after infection (**C**), percentage of eosinophils in peripheral blood leucocytes after infection (**D**), and number of mast cells in peritoneal exudate cells (PEC) after infection (**E**) of each genotype of mice are shown. n=1–5 mice per genotype (see **Supplemental Table 1**) from eight independent experiments. Purple, blue, red, green, black, and white lines, bars and dots represent CC012, CC015, CC027, CC061, C57BL/6J and BALB/c mice, respectively. Barplots with error bars for summary graphs indicate means ± SDs, and each dot indicates one mouse. Dashed lines in violin plots indicate 25th and 75th quartiles and median.

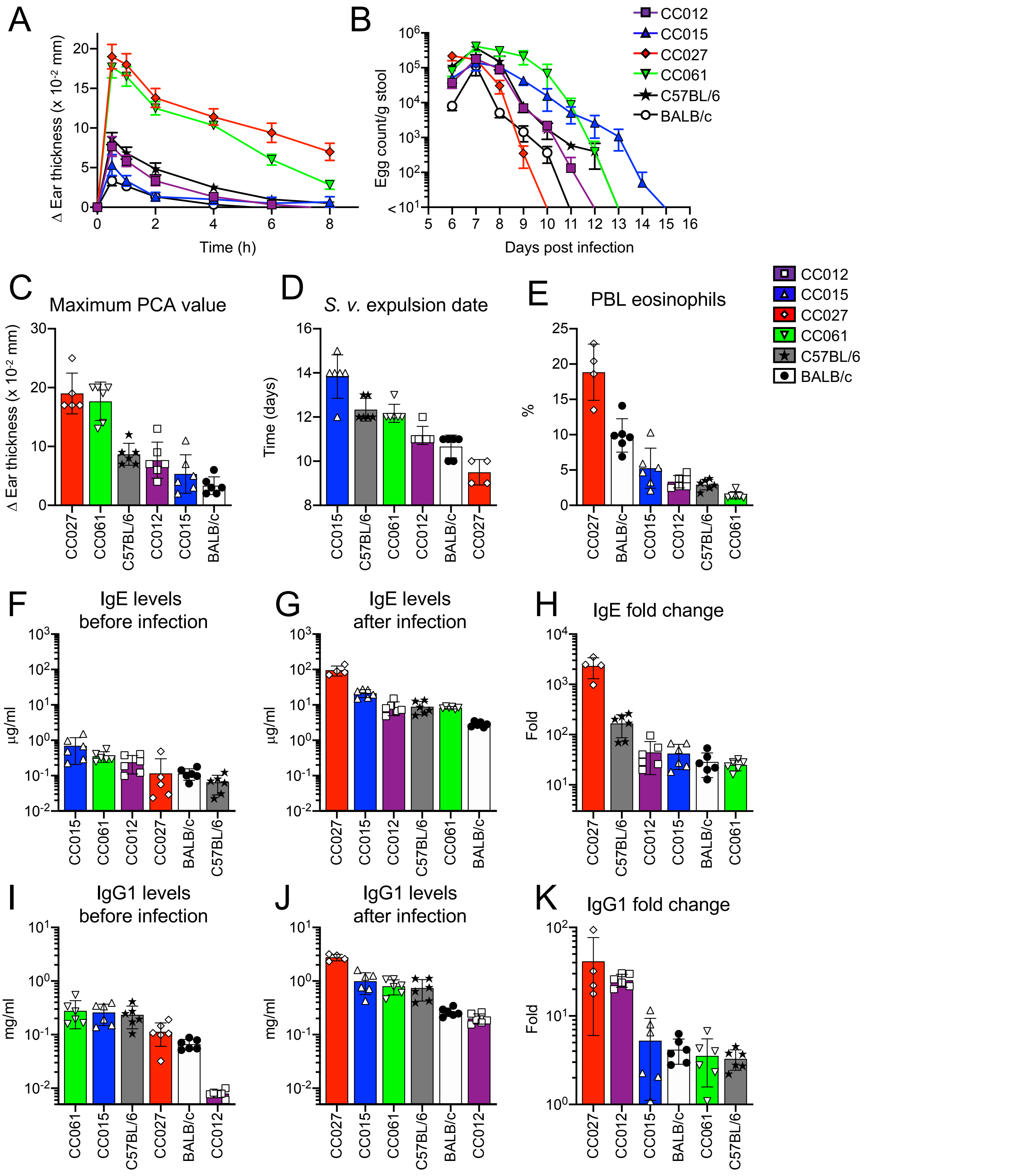

Supplemental Figure 4. PCA intensity and immunological parameters were evaluated before and after *S. v.* infection in CC, C57BL/6J and BALB/c mice.

CC012, CC015, CC027, CC061, C57BL/6J and BALB/c mice were treated as in **Figure 1A**. (**A**) PCA reaction. Mean changes (Δ) in ear thickness over time after intravenous injection in each genotype of mice are shown. (**B**) *S. v.* infection. Mean *S. v.* egg numbers excreted after worm inoculation in each genotype of mice are shown. (**C to K**) Summary graphs of the maximum values of PCA (**C**), *S. v.* expulsion date (**D**), percentage of eosinophils in peripheral blood leucocytes after infection (**E**), serum IgE levels before (**F**) and after (**G**) *S. v.*-infection, and the IgE fold change after infection (**H**), serum IgG1 levels before (**I**) and after (**J**) *S. v.*-infection, and the IgG1 fold change after infection (**K**). All samples in E, G and J were obtained three days after the mice had stopped producing *S. v.* eggs. Purple, blue, red, green, black and white lines and bars represent CC012, CC015, CC027, CC061, C57BL/6J and BALB/cJ, respectively. Barplots with error bars indicate means ± SDs, and each dot indicates one mouse. n=4 for CC027 and n=6 for other genotypes from three independent experiments.

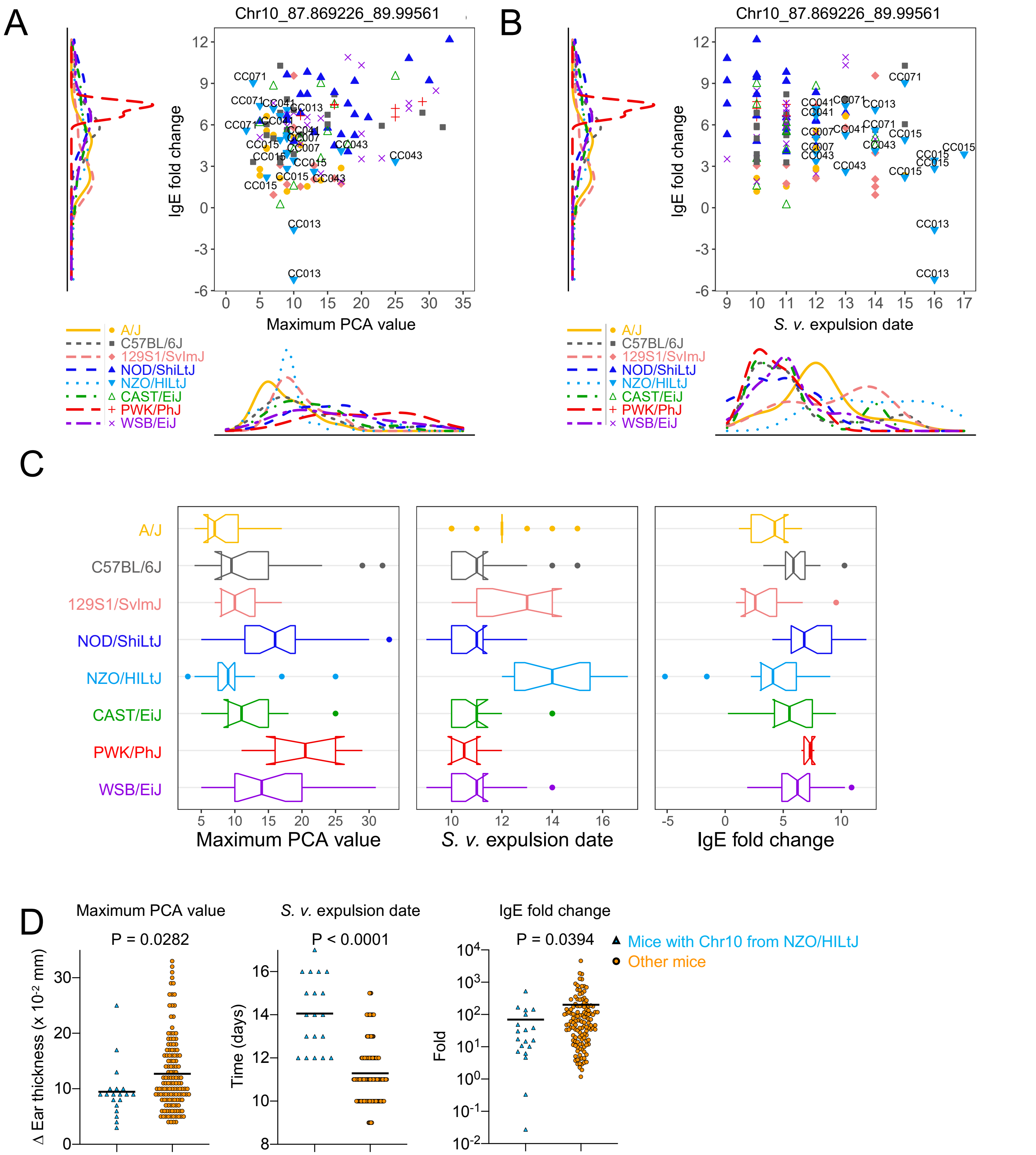

Supplemental Figure 5. Chromosome 10, region 79.4–94.0 Mb, from NZO/HILtJ associates with weaker MC responses in CC mice.

(**A and B**) Inter-correlation among maximum PCA value, *S. v.* expulsion date, and IgE-fold change in CC mice as in **Figure 2**. The founder strain of each CC mouse's chromosome 10, 87.9–90.0 Mbp are indicated in eight different colors (yellow; A/J, brown; C57BL/6J, pink; 129S1/SvImJ, dark blue; NOD/ShiLtJ, light blue; NZO/HILtJ, green; CAST/EiJ, red; PWK/PhJ, purple; WSB/EiJ). (**C**) CC mice were classified into eight groups according to the founders of their chromosome 10, 87.9–90.0 Mbp. Notched boxplots of maximum PCA value, *S. v.* expulsion date, and IgE-fold change of each group are shown. (**D**) Mice were divided into two groups according to their chromosome 10, 87.9–90.0 Mbp being from NOD/ShiLtJ (light blue) or not (orange). Dotplots of maximum PCA value, *S. v.* expulsion date, and IgE-fold change of each group are shown. Each dot indicates one mouse and the bars indicate the means. P values were determined by Student's *t* test.

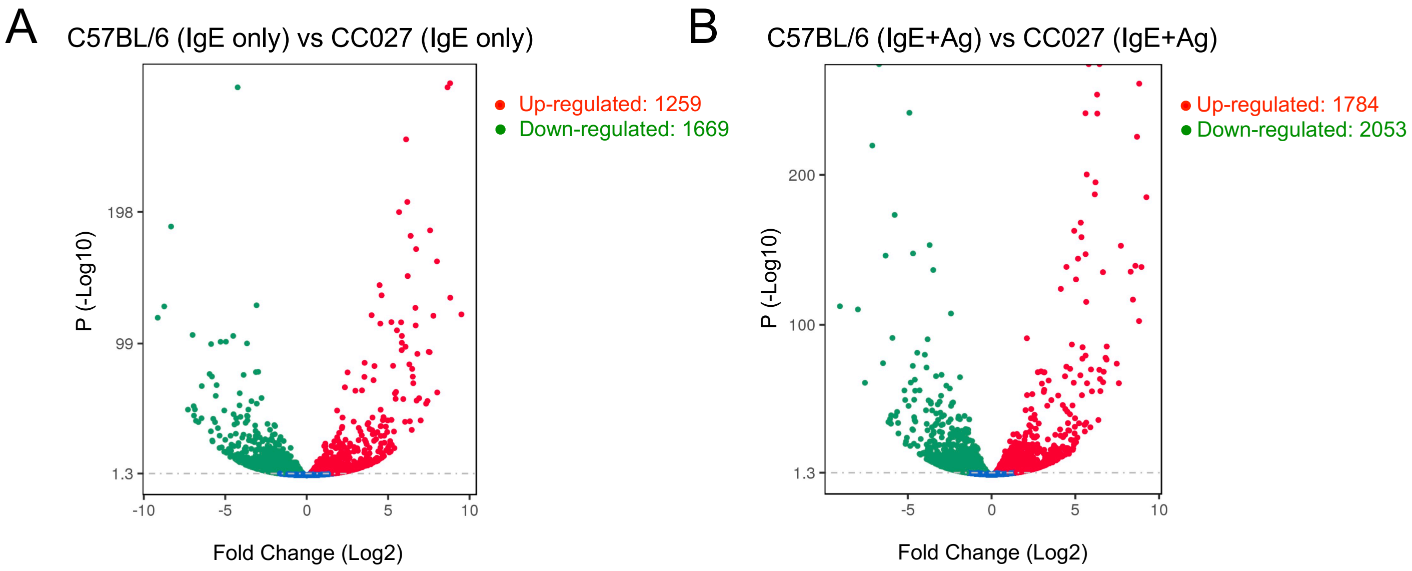

Supplemental Figure 6. BMCMCs of CC027 and C57BL/6J mice differ in RNA expression profiles.
BMCMCs from CC027 and C57BL/6J mice were prepared and stimulated as in **Figure 4.** MCs were sensitized with mouse anti-DNP-IgE mAb (1 μg/mL) overnight, then stimulated with (IgE+Ag) or without (IgE only) DNP-HSA (20 ng/mL) for 1 hour. Volcano plots of differentially expressed genes between BMCMCs from CC027 and C57BL/6J mice stimulated without (**A**; IgE only) or with (**B**; IgE+Ag) DNP-HSA.

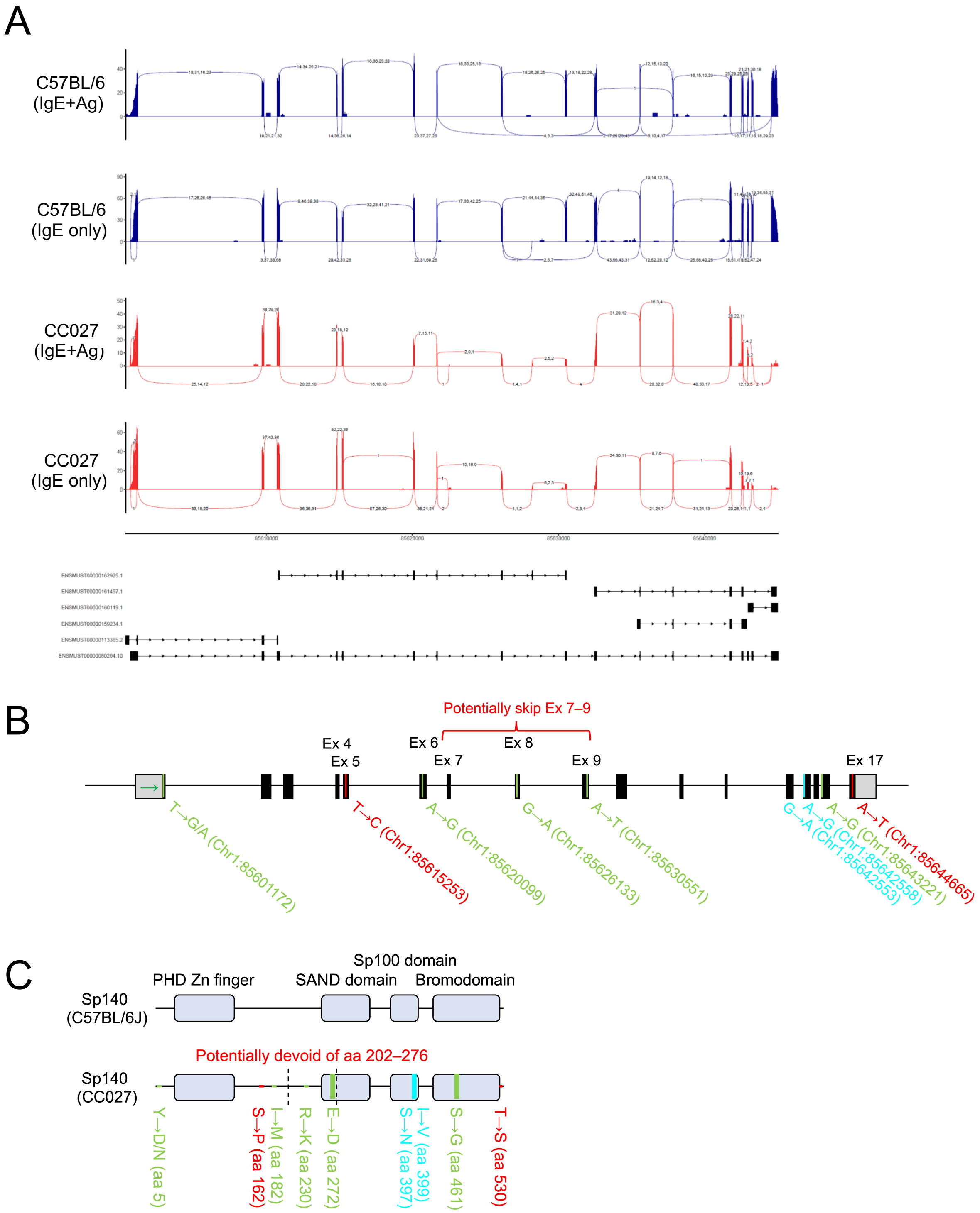

Supplemental Figure 7. CC027 mice express *Sp140* devoid of Ex 7–9.
(**A**) Sashimi plots showing alternative splicing pattern for the entire *Sp140* in C57BL/6J (blue) and CC027 (red) mice. (**B**) Schematic representation of the mouse *Sp140* gene. Nine missense SNPs (red; NOD/ShiLtJ-specific SNPs, green; SNPs not specific for NOD/ShiLtJ, light blue, newly identified SNPs) on CC027 *Sp140* are indicated. (**C**) Schematic representation of the mouse Sp140 protein. The expected Sp140 protein expressed in C57BL/6J and CC027 mice, and the point mutations in CC027 mice caused by SNPs, are shown.

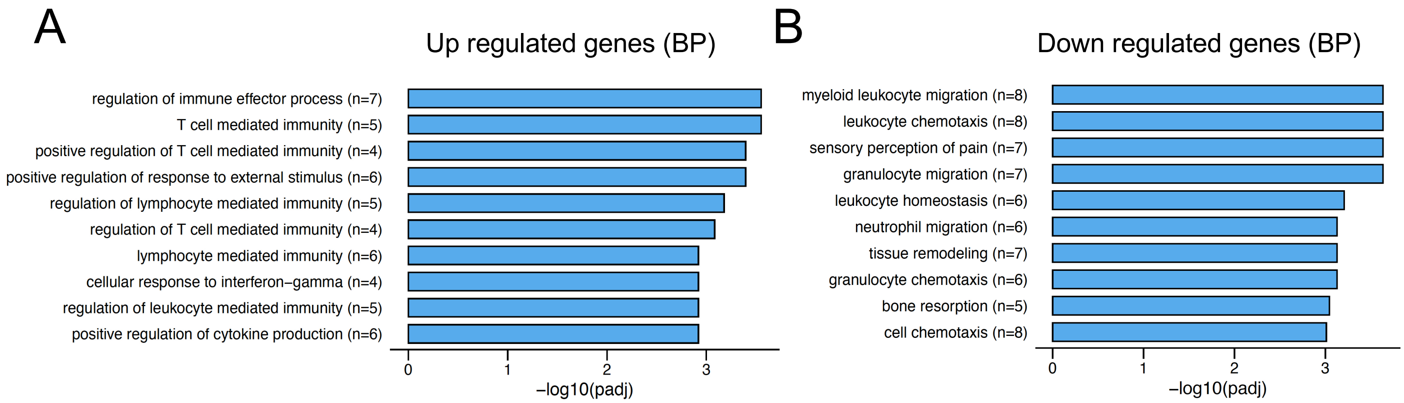

Supplemental Figure 8. *Sp140*-controlled genes contribute to immune-related processes in CC027 BMCMCs. GO biological processes of up- (**A**) and down- (**B**) regulated *Sp140*-controlled genes (mouse macrophages) in non-stimulated CC027 BMCMCs. Genes extracted in **Figure 6I** were subjected to GO pathway analysis.

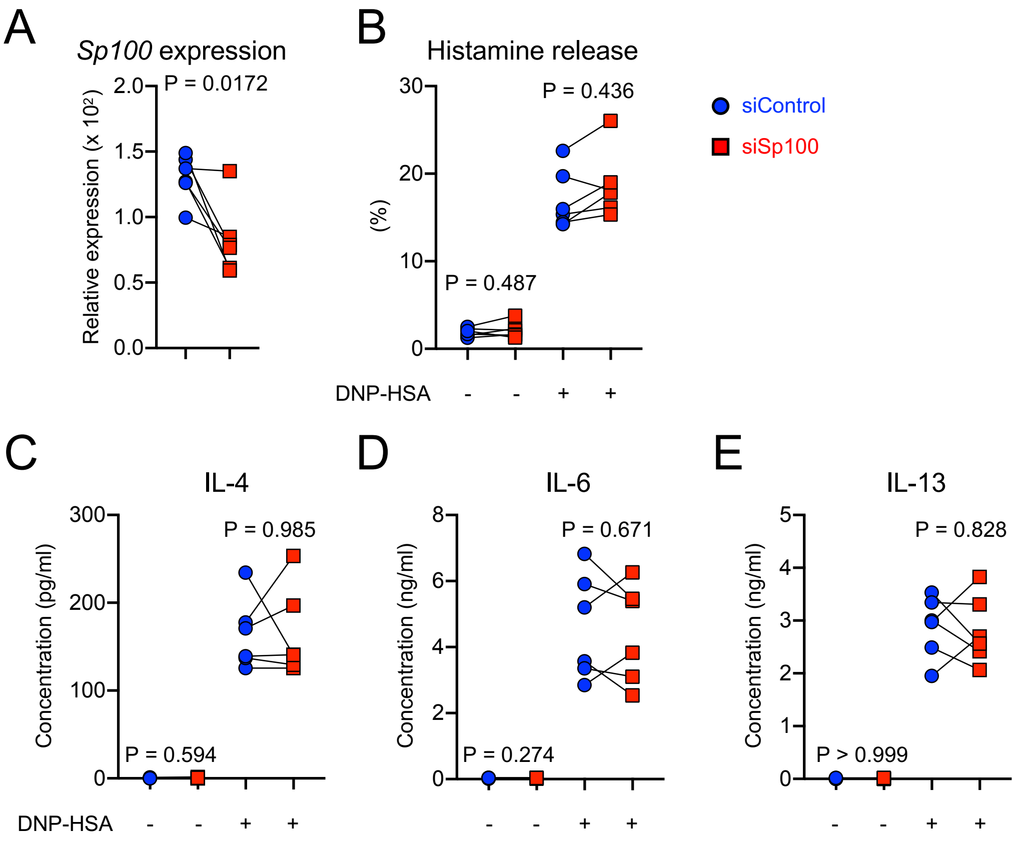

Supplemental Figure 9. *Sp100* is dispensable for IgE-dependent MC responses.
Bone marrow cells from C57BL/6J mice were cultured with IL-3 for 6–7 weeks to generate BMCMCs. Control siRNA or siRNA against *Sp100* was transfected into the cells. (**A**) mRNA level for *Sp100* twenty-four hours after the siRNA transfection was determined by quantitative PCR. The *Sp100* levels are indicated as relative to *Actb*. (**B**) siRNA-transfected cells were sensitized with mouse anti-DNP-IgE mAb (1 μg/mL) overnight, then stimulated with DNP-HSA (10 ng/mL) for 1 hour. Histamine content in cell lysates and culture supernatants was determined by ELISA. Histamine release rates are indicated as % release (supernatants) out of total histamine contents of the cells (cell lysate plus supernatant). (**C to E**) siRNA-transfected cells were stimulated as in (**B**) but for 6 hours. The levels of IL-4 (**C**), IL-6 (**D**) and IL-13 (**E**) in the culture supernatants were determined by ELISA. Each dot indicates different biological replicates. n=6 pooled from two independent experiments. P values were determined by paired Student's *t* test.
